# Delivery mechanism can enhance probiotic activity against honey bee pathogens

**DOI:** 10.1038/s41396-023-01422-z

**Published:** 2023-06-14

**Authors:** Brendan A. Daisley, Andrew P. Pitek, Christina Torres, Robin Lowery, Bethany A. Adair, Kait F. Al, Bernardo Niño, Jeremy P. Burton, Emma Allen-Vercoe, Graham J. Thompson, Gregor Reid, Elina Niño

**Affiliations:** 1 Department of Molecular and Cellular Biology, University of Guelph, Guelph, ON N1G 2W1 Canada; 2 Department of Microbiology & Immunology, University of Western Ontario, London, ON N6A 5B7 Canada; 3 Department of Biology, The University of Western Ontario, London, ON N6A 5B7 Canada; 4 Department of Entomology and Nematology, University of California, Davis, Davis, CA 95616 USA; 5 Agricultural Research Service, United States Department of Agriculture, Davis, CA 95616 USA; 6 University of California Agriculture and Natural Resources, Oakland, CA 95618 USA

**Keywords:** Microbiome, Metagenomics, Applied microbiology

## Abstract

Managed honey bee (*Apis mellifera*) populations play a crucial role in supporting pollination of food crops but are facing unsustainable colony losses, largely due to rampant disease spread within agricultural environments. While mounting evidence suggests that select lactobacilli strains (some being natural symbionts of honey bees) can protect against multiple infections, there has been limited validation at the field-level and few methods exist for applying viable microorganisms to the hive. Here, we compare how two different delivery systems—standard pollen patty infusion and a novel spray-based formulation—affect supplementation of a three-strain lactobacilli consortium (LX3). Hives in a pathogen-dense region of California are supplemented for 4 weeks and then monitored over a 20-week period for health outcomes. Results show both delivery methods facilitate viable uptake of LX3 in adult bees, although the strains do not colonize long-term. Despite this, LX3 treatments induce transcriptional immune responses leading to sustained decreases in many opportunistic bacterial and fungal pathogens, as well as selective enrichment of core symbionts including *Bombilactobacillus*, *Bifidobacterium*, *Lactobacillus*, and *Bartonella* spp. These changes are ultimately associated with greater brood production and colony growth relative to vehicle controls, and with no apparent trade-offs in ectoparasitic *Varroa* mite burdens. Furthermore, spray-LX3 exerts potent activities against *Ascosphaera apis* (a deadly brood pathogen) likely stemming from in-hive dispersal differences, whereas patty-LX3 promotes synergistic brood development via unique nutritional benefits. These findings provide a foundational basis for spray-based probiotic application in apiculture and collectively highlight the importance of considering delivery method in disease management strategies.

## Introduction

Managed honey bee (*Apis mellifera*) populations are leveraged globally to support adequate pollination of food crops and contribute upwards of US$225 billion annually to agricultural economies [1]. However, maintaining the health of these crucial insects (as well as wild pollinators) within agroecosystems is exceedingly difficult in the face of widespread pesticide use and diminished floral diversity, which together exacerbates susceptibility to infectious disease [2]. Contributing most to the unsustainable colony losses reported in the US over recent years are ectoparasitic mites and a broad range of bacterial, fungal, and viral pathogens. Despite vigilant efforts of beekeepers in applying antibiotics (e.g., oxytetracycline, fumagillin) and other hive chemicals (e.g., varroacides) as prophylactic measures against disease outbreaks, there has been no clear progression toward improvement with annual colony losses averaging ~39.4% (more than twice the sustainable limit of 15%) over the last decade [3]. Moreover, mounting evidence suggests these efforts could have counterproductive outcomes by promoting antibiotic resistance and inadvertently damaging the honey bee gut microbiota—a critical health component orchestrating overall colony-level survival via effects on digestion [4], immune regulation [5], and overwintering success [6]. In accordance with these realizations, there is a growing interest in supporting honey bee health via microbiota modulation strategies, and relatedly, how probiotics can be used as sustainable methods of disease control [7].

While fungal communities remain poorly described, bacterial communities associated with honey bees have been intensively studied [8, 9]. Regarding the bacterial component, honey bees possess a stable “core” gut microbiota (present in nearly all healthy individuals worldwide) that is dominated by several lactic acid-producing genera (*Bombilactobacillus*, *Lactobacillus*, and *Bifidobacterium*) and select proteolytic genera (*Gilliamella* and *Snodgrassella*) [10]. Other bacteria such as *Apilactobacillus*, *Frischella*, *Commensalibacter*, and *Bartonella* spp. found at lower abundance can also play important health roles [11]. General consensus suggests that low levels of lactic acid bacteria (LAB) and high levels of proteobacteria are a sign of gut dysbiosis and deteriorated health status in honey bees [12]. This trend is consistently observed in social animals including humans and mice, despite divergent physiology and gut microbiota composition [13]. In vitro experiments have shown that LAB (endogenous and exogenous strains) exhibit the strongest inhibitory properties against many important larval pathogens including *Paenibacillus larvae* (bacterial agent of American Foulbrood disease), *Melissococcus plutonius* (bacterial agent of European foulbrood), *Ascosphaera apis* (fungal agent of Chalkbrood disease), and *Aspergillus niger* (fungal agent of Stonebrood disease) [14–16]. Despite variable mechanisms of action, oral supplementation of inhibitory LAB strains (via larval food inoculation) under laboratory conditions have consistently been shown to improve survival against the aforementioned pathogens. In contrast, field studies have shown less reproducibility. For example, a 13-strain mixture of honey bee-specific lactobacilli failed to prevent American foulbrood [17] despite showing strong in vitro activity against *P. larvae* [14]. Although the complex social behaviors of honey bees may contribute to the inconsistencies observed between laboratory and field study findings, a simpler explanation could be related to methodological differences in delivery method (see review [18]).

There has been very limited investigation into how delivery of probiotics to the hive can impact their efficacy, with predominantly two main strategies having been tested: sucrose syrup suspension and pollen patty inoculation. Sucrose syrup suspensions remain the most popular method based on ease of application, but osmotic stress can induce rapid cell lysis of many bacteria (e.g., >90% reduction in LAB cell viability within 96 h at 30 °C [19]) making it a poor delivery vehicle in most instances. In contrast, pollen patty-based methods offer a more suitable system (viable bacteria shown to reach their intended targets of the honey bee intestinal tract [20, 21]) while also contributing nutritional benefits that support overall health [22]. A major drawback to either method is that only adult bees directly consume the product, meaning that distribution throughout the brood chamber (where many larval pathogens exist) is primarily reliant on nutrient flux between nurse bees (which directly consume the product) and larvae (which are fed by nurse bees via trophallaxis) in the hive [21]. One way to ensure physical dispersion through the brood chamber could be through dusting of hive frames with freeze-dried bacteria [23]. From a practical perspective though, this would be time-consuming alongside concerns of clumping and uneven distribution. We herein explore a potentially promising alternative, namely a spray-based application of beneficial bacteria suspended in an isotonic solution. Spray-based probiotic applications have broad relevance with recent studies showing success against clinically relevant viral infections [24] as well as in the prevention of white-nose syndrome in the little brown bat (*Myotis lucifugus*) [25]. Notably, a spray-based approach could largely eliminate any viability concerns (e.g., bacterial cells are estimated to be viable on a scale of months to years in phosphate-buffered saline [26]) and has potential to enable high-throughput dispersal of bacterial inoculum throughout an entire hive.

In our previous work, we demonstrated that a patty-based hive supplement comprising a three strain LAB consortium (*Lactiplantibacillus plantarum* Lp39, *Lacticaseibacillus rhamnosus* GR-1, and *Apilactobacillus kunkeei* BR-1; herein referred to as LX3) could strongly suppress *P. larvae* burden in both symptomatic and asymptomatic colonies, as well as induce beneficial effects on adult immune and microbiota systems [20, 27]. Based on these multifaceted mechanisms and general improvement of host constitution, LX3 could be expected to increase resistance toward a broad range of infectious diseases. The spray-based delivery system we developed in the current study was used to compare LX3 application with the previously established patty-based (“BioPatty”) system for purposes of improving overall colony-level health in a pathogen-dense region of California. A 24-week longitudinal field study was performed with analyses focused on the systems-level characterization of entire bacterial and fungal communities (rather than single pathogens of interest) to provide a more complete understanding of the microbial ecology of honey bee health and disease.

## Methods

### Bacterial strains and culture conditions

The three lactobacilli strains used in this study were *L. plantarum* Lp39 (American Type Culture Collect [ATCC] 14917), *L. rhamnosus* GR-1 (ATCC 55826), and *Api. kunkeei* BR-1 (a honey bee gut-derived isolate from a healthy hive [27]). Routine culturing of these strains was performed under microaerophilic conditions at 37 °C using de Man, Rogosa, and Sharpe (catalog number: 288130, BD Difco) broth or agar supplemented with 10 g/l D-fructose (catalog number: F-3510, Sigma-Aldrich; MRS-F). Harvest of bacterial cells was similar for patty- and spray-based LX3 treatments. Briefly, fresh streak plates were incubated overnight and then a single colony of each strain was used to inoculate multiple broth cultures (i.e., LX3 strains were grown separately to ensure standardized dose) that were subsequently incubated at 37 °C for 24 h using sterile 50 ml polypropylene conical tubes (catalog number: 339652, Thermo Scientific; MRS-F filled to 50 ml, lids tightly closed). Following incubation, bacterial cells were centrifuged at 5000 × *g* for 10 min (4 °C), washed once with 0.01 M PBS, centrifuged again at 5000 × *g* for 10 min (4 °C), resuspended in 0.01 M PBS, and then strains were mixed together in a final concentrated volume of 4 ml 0.01 M PBS at equal cell densities (i.e., the mixture contained 5 × 10^10^ colony forming units [CFU] of each LX3 strain). Ultimately, all hives treated with LX3 (using either patty- or spray-based methods) received the same total amount of bacterial cells (i.e., 5 × 10^10^ CFU of each LX3 strain) at W0 and W2 timepoints.

### Recipe for patty- and spray-based LX3 treatments

For patty-based treatments, the base nutritional matrix of each 250 g patty consisted of the following standard pollen substitute ingredients: 28.5 g of soy flour, 74.1 g of granulated sucrose, 15.4 g of debittered brewer’s yeast, 132.1 g of a 2:1 (w/v) simple sucrose-based syrup solution. For LX3 patties, 4 ml of the concentrated LX3 suspension in 0.01 M PBS was added followed by vigorous stirring to obtain a homogenous infusion at a final concentration of 2 × 10^8^ CFU/g for each strain. For vehicle patties, 4 ml of sterile 0.01 M PBS was added instead. Each patty was poured in between two sheets of wax paper (30 cm × 45 cm) and immediately supplemented to hives (placed on top of brood chamber) within a 24 h period.

For spray-based treatments, the 4 ml concentrated LX3 suspension was added to 28 ml of 0.01 M PBS in a sterile spray bottle to obtain a diluted concentration of 1.56 × 10^9^ CFU/ml per strain. We determined that the nozzles of the bottles discharged 2 ml per spray. Accordingly, 32 ml of the LX3-containing suspension was administered to the hive via 16 standardized spray actions (2 × 2 ml front and back of each brood frame, for 8 brood frames per hive). The same spray sequence was used for the vehicle spray, but with 32 ml sterile 0.01 M PBS added to a sterile spray bottle instead. Supplementation with probiotic treatments occurred at W0 and W2 timepoints (after hive measurements and sampling were completed in both instances) and hives were monitored thereafter until W24.

### Apiary set-up, experimental overview, and sampling procedure

Field trials were performed on managed honey bee colonies headed by naturally mated queens of mixed Italian background (*A. m. ligustica*). For the duration of the study, colonies were located at two experimental apiaries (LTRAZ and RAPTOR) approximately four miles apart near the University of California, Davis (Davis, California, United States). A total of 33 colonies (17 located in LTRAZ and 16 located in RAPTOR) were used for this study and were housed in standard Langstroth hives that were elevated ~36 inches above ground level using wooden hive stands. Hives were randomly distributed across the two sites (to account for spatial confounders) and divided into five experiments groups: (1) no treatment control (NTC; *n* = 7 hives), (2) pollen patty vehicle control (P; *n* = 7 hives), (3) pollen patty containing LX3 (P + LX3; *n* = 7 hives), (4) spray vehicle control (S; *n* = 6 hives), and (5) spray containing LX3 (S + LX3; *n* = 6 hives). The relevant hives of interest were treated at the start of the study (W0) and again 2 weeks later (W2), followed by a 22-week monitoring period during which none of the groups received any further treatment.

Prior to start of the experiment, all colonies were split and equalized to contain approximately equal number of frames of bees, brood, and food stores (pollen and nectar/honey) and were requeened with age-matched queens from the same queen producer (Jackie Park-Burris Queens, Inc., Palo Cedro, CA). Hives were provided a queen excluder and supered with a fresh 10-frame deep box to allow for quantification of honey stores. Colonies were managed according to standard practices suitable for the dry Northern California climate often lacking natural forage throughout the year. Specifically, all hives were provisioned with carbohydrate supplement (ProSweet syrup, Mann Lake Ltd., Woodland, CA) prior to project start (04/12 and 04/23) and at W2 and W4, and all hives were provisioned with protein supplement (Bee-Pro Patties+, Mann Lake, LTD., Woodland, CA) at W6 and W13.

Sampling of adult nurse bees (found in close physical proximity of larvae in the brood chamber) occurred mid-day at W0, W2, W4, W6, W8, W12, and W24 timepoints and was achieved by gently dragging a 50 ml conical tube (catalog number: 339652, Thermo Scientific) along the surface of the most central brood frame in a hive until the container was approximately half full (~50 bees in total). Samples were then flash frozen in the field using liquid nitrogen and subsequently stored at −80 °C until downstream DNA/RNA extraction steps. All sensible precautions were taken to prevent cross-contamination of LX3 strains and potential pathogens between hives; new sets of sterile latex gloves were used for each hive, hive tools were flame sterilized between opening of hives, and a strict group-wise sampling order was enforced (e.g., NTC → S → P → S + LX3 → P + LX3).

### Estimation of colony sizes

Frames of bees were determined via routine methods at W0, W2, W4, W6, W8. W12, W16, W20, and W24 timepoints. Under the assumption that each deep brood frame (43.1 cm × 20.3 cm) can hold ~2430 adult workers [28], total colony size was calculated as total frame of bees × 2430 workers per frame.

### Determination of capped brood area

To assess how treatments impacted the reproductive performance of queens, coverage of capped brood cells on hive frames were measured at W0, W2, W4, W6, W8, W12, and W24 timepoints. Digital photographs were taken for both sides of the center three frames (labeled 3, 4, and 5 to ensure consistent order) in the brood chamber of each hive (i.e., 6 photos per hive, per timepoint). Subsequently, capped brood cells were quantified from a total of 1386 photographs collected during the field study.

### Determination of ectoparasitic mite loads

*Varroa destructor* was the only ectoparasite detected in the cohort of honey bees assessed in this study. Hive burden of this parasite was determined monthly at W0, W4, W8, and W12 timepoints. Routine alcohol wash was used to measure *V. destructor* burden. Briefly, a half-cup measuring device was used to collect ~300 adult bees from active brood areas, the bees were placed in a jar containing 70% ethanol, shaken vigorously for 1 min, and then dislodged mites were collected (via size-based exclusion through a mesh strainer), counted, and recorded. Raw counts were converted to percent mite population (i.e., number of mites per 100 bees) for interpretation purposes.

### TRIzol-based dual extraction of RNA and DNA

Adult honey bee samples were thawed from −80 °C and dissected in a cold room at 4 °C. Samples were visually screened for rectums yellow-to-orange in appearance (indicating a pollen-based diet of nurse bees) whereas rectums with a translucent appearance (indicating a nectar-based diet of foragers) were discarded. The dissected hindguts and heads of selected samples were then pooled in biological triplicate and homogenized in TRIzol (Invitrogen) by bead beating, followed by RNA extraction via the manufacturer’s instructions. From the remaining TRIzol interphase layer, DNA was extracted using a back-extraction buffer consisting of 4 M guanidine thiocyanate, 50 mM sodium citrate dihydrate, and 1 M Tris base, as previously described [20]. Quality of RNA and DNA was assessed using a microvolume spectrophotometer (DS-11 Spectrophotometer; DeNovix). Samples with A260/280 absorbance ratios between 1.8 and 2.2 for RNA and between 1.6 and 2.0 for DNA were considered for further analyses.

### Amplicon library construction and sequencing parameters

Targeted amplification of the V3-V4 region of the 16S rRNA gene was achieved using the established Bakt_341F (5’-CCTACGGGNGGCWGCAG-3’) and Bakt_805R (5’-GACTACHVGGGTATCTAATCC-3’) primer set, shown to be optimal for characterization of honey bee-associated bacterial communities [29]. For fungal community profiling, the ITS1f (5’-CTTGGTCATTTAGAGGAAGTAA-3’) and ITS2 (5’-GCTGCGTTCTTCATCGATGC-3’) modified primer set was used as specified in the Earth Microbiome Project (EMP) ITS amplicon protocol (https://earthmicrobiome.org/protocols-and-standards/its). Full primer constructs including 12-mer GOLAY barcodes and Illumina adapters are provided in Supplementary Data 1A, B). Initial amplicon generation was similar for both libraries and was achieved by adding 2 µl of sample DNA (5–50 ng/µl) to a 96-well 0.2 ml PCR plate containing 20 µl of pre-mixed forward and reverse primers (both at working concentrations of 1.6 µM). Next, 20 µl of GoTaq 2X Colorless Master Mix (Promega) was added to each well (final volume of 42 µl) and plates were sealed using PCR-grade adhesive aluminum foil. PCR steps were performed using a Prime Thermal Cycler (Technie) with 105 °C lid temperature with the following reaction conditions: 95 °C for 3 min, followed by 30 cycles of 95 °C for 1 min, 52 °C for 1 min, and 72 °C for 1 min, followed by a final extension step at 72 °C for 5 min, and then amplicons were stored at −20 °C until further processing. Processing of amplicon libraries was conducted at the London Regional Genomics Centre (Robarts Research Institute, London, ON, Canada) in which amplicons were quantified using PicoGreen (Quant-It; Life Technologies, Burlington, ON, Canada), pooled at equimolar ratios, and sequenced on the MiSeq platform (Illumina) adapted for 2 × 300 bp paired-end V3 chemistry.

### General processing of sequencing datasets

All code and scripts used for processing sequence data have been uploaded to a repository and made publicly available at https://github.com/bdaisley/LX3CA1. Briefly, sequencing FASTQ files for both datasets were demultiplexed using Cutadapt (v3.4) with default settings for combinatorial dual indexes and 0% error tolerance. Forward and reverse sequence reads were then dereplicated, denoised, and merged via the DADA2 (v1.16) pipeline to infer exact (i.e., 100% identity, not clustered) amplicon sequence variants (ASV) from the datasets (quality profiles shown in Supplementary Fig. 1). For the 16S rRNA dataset, a total of 6,040,822 filtered reads remained following quality assurance steps and after denoising, a total of 848 unique bacterial ASVs were identified. For the ITS dataset, a total of 6,604,619 filtered reads remained following quality assurance measures and after denoising, a total of 3387 unique fungal ASVs were identified (Supplementary Data 1C). ASV read counts were left in their unadulterated state for analysis (i.e., read counts were not adjusted using predicted genomic copy number differences) based on the latest best-practice recommendations [30]. Zero counts were adjusted via a Bayesian-based multiplicative zero-replacement method using the *cmultiRepl* function of the zCompositions (v1.3.4) package in R (v4.0.1) and count tables were center log-ratio (CLR) transformed prior to compositional comparisons [31]. Raw FASTQ files were uploaded to the NCBI Sequence Read Archive and are accessible under BioProject IDs PRJNA856263 (16S rRNA dataset) and PRJNA856341 (ITS dataset).

### Taxonomic annotation of bacterial and fungal sequencing reads

To assess the biological relevance of sequencing data, bacterial ASVs were assigned taxonomy using the *idtaxa* function of the DECIPHER (v2.20.0) package in R with the BEExact (v2021.0.2; https://github.com/bdaisley/BEExact) pre-trained V3V4 database [29], which enabled species-level classification for 97.9% of sequences (including “bxid” annotations of taxa lacking formal nomenclature; global pairwise identity scores in Supplementary Data 1D). Fungal ASVs were assigned taxonomy in a similar manner using the UNITE database (v8.3-RefS; 10.15156/BIO/1280049), which enabled species-level classification for 35.17% of sequences (Supplementary Data 1E). To enable further predictive analysis with unclassified ASVs (belonging to bacterial and fungal dark matter) we applied phylogenetically-consistent placeholder names based on closest identities with known species representatives as previously described [29]. Briefly, all unclassified ASVs were cross-referenced at their lowest common ancestor (LCA) rank using NCBI’s Bacterial 16S rRNA and Fungal ITS RefSeq Targeted Loci project (https://ftp.ncbi.nlm.nih.gov/refseq/TargetedLoci) databases, which contain curated reference sequences from type strain material. Subsequently, pairwise distance matrices between ASVs were used to generate probabilistic de novo taxonomic groupings (via a greedy-clustering algorithm) at each unclassified taxonomic rank based on previously established thresholds [32, 33]. To differentiate between bacterial and fungal placeholder names, sequences were annotated as either “bSV-###” or “fSV-###”, respectively. Scripts used for generating de novo taxonomy are available at https://github.com/bdaisley/LX3CA1.

### Microbial diversity, differential abundance, and correlation analyses

Alpha diversity metrics were calculated using the microbiome (v.1.14.0) package in R. Unconstrained (PCoA) and constrained (db-RDA) analyses of beta diversity were determined using the phyloseq (v1.36.0) and philr (v1.18.0) packages in R. The “deicode” plugin for QIIME2 (v2021.4) was used to generate robust-CLR (rCLR) ordination plots. Treatment group comparisons of beta diversity metrics were calculated via PERMANOVA tests with the “adonis2” function of the vegan (v2.5.7) package in R (permutations = 9999, method = “euclidean”). To assess pairwise multilevel comparisons, the “pairwise.adonis2” function of the pairwiseAdonis package (v0.4) in R was used with apiary location (i.e., LTRAZ or RAPTOR) and hive identity set as strata factors in the model block design. In addition, the recently developed Wd*-test (a robust alternative for distance-based multivariate analysis of variance [34]) and associated Tw2-tests for multiple comparisons were implemented for consensus purposes. Differential abundance tests were performed using a generalized linear mixed model approach with the MaAsLin2 package (v1.7.3) in R. Briefly, CLR-transformed relative abundance values were used as input values with timepoint, treatment group, capped brood area, and colony size as fixed effects; the latter factors were determined as potential confounders in the metadata using the envfit function of the vegan package (v2.5.7) in R (see Supplementary Data 1F). Relatedly, change point analyses at the phylum-level were determined using a spline-based permutation method via the sliding_spliner function of the splinectomeR package (v0.1.0) in R. For host immune-microbe correlation analyses, the aldex.corr function of the ALDEx2 package (v1.24.0) in R was utilized with Ct values (for host gene expression, normalized to the Rp5S endogenous control) and CLR-transformed relative abundances used as input values.

### Co-occurrence network analysis

Networks were constructed and analyzed using the NetCoMi package (v1.0.2.9001) in R. A recommended prevalence cut-off of 50% (i.e., bacterial and fungal ASVs were only included as inputs if they were present in least half of all samples) was utilized to address sparsity limitations of sequencing data. Remaining zeroes in the datasets were addressed using the multRepl function (method = ”CZM”) prior to normalization via CLR-transformation [31], and then networks were produced via the netConstruct function (multiple comparisons corrected using options: measure = ”spearman”, nboot = 1000, adjust = ”adaptBH”, alpha = 0.05). Global network and largest connected component (LCC) properties were determined using the netAnalyze and netCompare functions with default settings.

### qPCR-based measurements of immune gene expression and absolute microbial abundances

To determine immune gene expression in hindgut and mouthpart samples, a total of 1500 ng extracted RNA from each sample was reverse transcribed to cDNA using the High-Capacity cDNA Reverse Transcription Kit following manufacturer’s instructions (Applied Biosystems, catalog number: 4368813). RT-qPCR amplifications (10 µl each in technical triplicate) were performed with tenfold-diluted cDNA using the Power SYBR Green kit (Applied Biosystems) following manufacturer’s instructions. Oligonucleotide primers targeting immune genes and the bacteria of interest are listed in Supplementary Data 1G. As per MIQE guidelines, honey bee *α-tubulin* was used as an endogenous control gene as described in previous work [20]. To determine bacterial and fungal absolute abundances, qPCR amplifications reactions (10 µl each in technical triplicate) were performed with tenfold-diluted DNA (derived directly from TRIzol back extraction) using the Power SYBR Green kit (Applied Biosystems) following manufacturer’s instructions. The universal Bakt_341F/Bakt_805R and ITS1f/ITS2 primer sets (described in the sequencing section) were used to quantify total bacteria and fungi, respectively. All qPCR-based amplifications were performed in DNase- and RNase-free 384-well microplates using a QuantStudio 5 Real-Time PCR System (Applied Biosystems) and analyzed with associated QuantStudio Design and Analysis software. Relative immune gene expression was calculated using the 2^−ΔΔCt^ method [35]. Absolute microbial quantification was calculated using an experimentally-determined standard curve as described previously [20]. Using the honey bee *Rp5S* gene as a stable reference point for host DNA concentration, an average Ct value of 20.96 was experimentally determined to correspond to a 10 mg (wet-weight) hindgut sample after considerations of dilution and extraction efficiency factors). Accordingly, before performing calculations to estimate DNA copies of 16S rRNA and ITS, sample ΔCt_*Rp5S*_ (relative to a setpoint of 20.96) was used to standardize Ct values corresponding to bacterial and fungal loads, respectively (i.e., absolute abundance data assumes an average hindgut weight of 10 mg per individual).

### Molecular quantification of microscopic parasites

For simplicity, parasite loads in this section refer strictly to Deformed wing virus (DWV) and *Vairimorpha ceranae* (formerly known as *Nosema ceranae*). Hive burden of these parasites was determined monthly at W0, W4, W8, and W12 timepoints. For DWV and *V. ceranae*, burdens were quantified (via qPCR and species-specific primers) using RNA and DNA, respectively, extracted from nurse bee hindgut tissue to remain consistent with immune and microbiota analyses. In each case, three pooled hindgut samples were assessed from each hive per timepoint (*n* = 396 in total). Cycle threshold (Ct) values for DWV were normalized to the relative expression of honey bee *α-tubulin* (endogenous control; XM_391936) and then expressed as the change in (normalized) DWV load at W4, W8, and W12 relative to baseline at W0. Alternatively, Ct values for *V. ceranae* were converted to absolute abundance using an experimentally determined standard curve and then standardized to a hindgut wet-weight of 10 mg using the genomic DNA abundance of the honey bee *Rp5S* gene as a reference point (i.e., average Ct threshold of 20.96 for Rp5S approximately corresponds to a 10 mg hindgut after consideration of dilution and extraction factors; see bacterial and fungal quantification sections for further details).

### Amino acid measurements and meta-analysis comparisons

Nutrient analysis of pollen patty matrices was performed by SGS Laboratories (Mississauga, ON). Amino acid profiles were analyzed using official AOAC 994.12 methodologies for livestock feed analysis. For purposes of comparing the relative protein quality of LX3 fermented patties, essential amino acid composition for beebread (a mixture of pollen with nectar or organic honey) and 16 common pollen sources were derived from previous reports [36–38]. Principal component analysis (PCA) was performed using the *prcomp* function in R (v3.6.0) with amino acid concentrations (g/16g N) used as input values and data scaled to be zero centered using the center = TRUE option. PCA results were plotted using the *scatter3D* function (options: theta = −202, phi = 0, bty = “u”) of the “plot3D” (v1.4) package in R (v3.6.0).

## Results

### LX3 effects on colony-level health metrics are modulated by delivery method

**Fig. 1:**
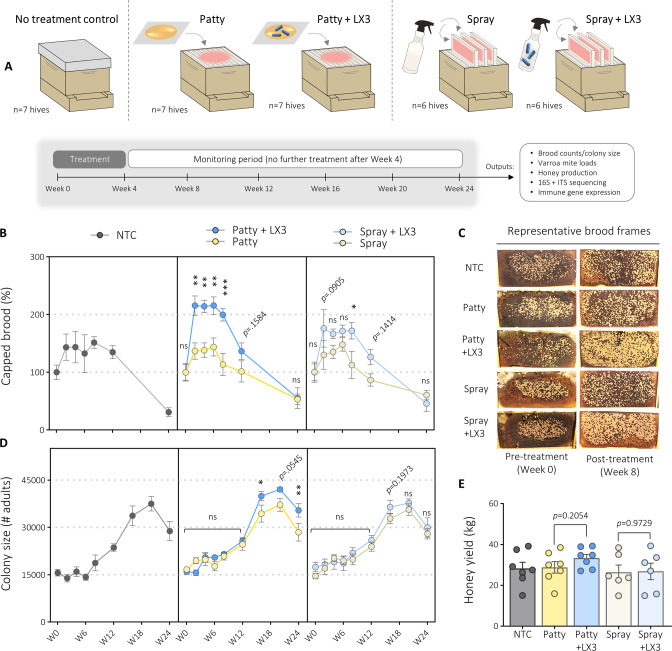
Delivery method alters LX3 effects on honey bee colony-level health metrics. **A** Schematic diagram outlining the experimental design. The 4 week treatment period consisted of two treatment doses that were administered at W0 and W2. No treatment occurred in any of the groups from W4 to W24. **B** Capped brood counts normalized to baseline values at W0 with individual hive estimates calculated from brood-box frame photos using semi-automated counting software. Data points depict mean ± SE for each group. Statistical comparisons within patty and spray groups are shown for three-way ANOVA with BH-adjusted *p* values. **C** Representative hive frame images used for enumeration of capped brood. Each hexagonal cell covered (capped) with wax and displaying a yellow-to-beige appearance depicts a single larva undergoing pupation to become an adult worker bee. **D** Estimated colony size of adult bees based on total hive frames containing adult bees. Data points depict mean ± SE for each group with statistics shown for three-way ANOVA with BH-adjusted *p* values. **E** Honey yield estimates at W12. Each data point depicts the weight of a honey super from a distinct hive, with bars representing the mean ± SE for each group shown. Statistics shown for unpaired two-tailed *t*-tests. *p < 0.05, **p < 0.01, ***p < 0.001, ns not significant.

A methodological overview of the study is provided in Fig. 1A. Firstly, to determine how the different treatments affected colony-level health outcomes, we quantified the coverage area of capped brood (established metric for assessing colony strength and reproduction [39]) from over 1000 hive frame photos taken during the study. Three-way analysis of variance (ANOVA) identified that timepoint (*F*_6,154_ = 30.42, *p* < 0.0001) and LX3 treatment (*F*_1,154_ = 41.23, *p* < 0.0001) had significant main effects, while vehicle type and LX3 treatment showed an interactive effect (*F*_1,154_ = 3.91, *p* = 0.0498; Fig. 1B, C). Pairwise comparisons showed that P + LX3 and S + LX3 treatments significantly increased capped brood between W2–W8 by magnitudes of over 100% and 70%, respectively, compared to vehicle controls (Fig. 1B). These findings are corroborated by previous work showing LX3 can rescue antibiotic-induced deficits in capped brood [20].

Complementing capped brood data, colony size (approximate number of adult bees per hive) also showed temporal fluctuations (Fig. 1D), which is consistent with expected seasonal variation [40]. Baseline colony sizes for the NTC group were smallest at W0 (~15,000 adults/hive) and largest at W20 (~38,000 adults/hive), with the P and S vehicle controls showing similar temporal dynamics. Pairwise comparisons showed larger colony sizes for P + LX3 relative to P at W16 (*p* = 0.0274), W20 (*p* = 0.0545), and W24 (*p* = 0.0051). S + LX3 treatment also demonstrated a similar but less pronounced trend toward increased colony size (Fig. 1D). Thus, early timepoint changes in capped brood corresponded proportionately with later timepoint colony size measurements for S + LX3 and P + LX3 groups (Fig. 1B–D). Single timepoint weighing of honey supers (additional boxes provided for honey storage used as an approximation of honey production) further showed a trend toward increased weight in the P + LX3 group relative to P group at W12, although the difference did not reach statistical significance (33.30 ± 1.86 vs. 28.78 ± 2.82 kg/hive, two-tailed *t*-test, *p* = 0.2054; Fig. 1E). Honey production nonetheless showed a positive correlation with changes in capped brood (*r*_s_ = 0.4380, *p* < 0.0122) and colony size (*r*_s_ = 0.5644, *p* = 0.0008, Spearman correlations; Supplementary Fig. 2), indicating potential colony performance benefits relevant to commercial beekeeping. These findings support that both patty- and spray-based LX3 treatment had positive overall impacts on colony status.

### Pollen patty nutritional benefits synergize with LX3 colony growth-promoting properties

Given *L. plantarum* (present in LX3) can increase plant protein digestibility by 92% [41], we sought to determine whether nutrient factors may explain the pronounced colony effects seen in the P + LX3 group (Fig. 1). In the laboratory, we incubated pollen patties (with or without LX3 added) for 1 week under simulated hive conditions and found that LX3 could greatly improve amino acid profiles. Specifically, 11 amino acids increased by 2–20% from LX3 addition (Supplementary Fig. 3 and Supplementary Data 1H). A meta-analysis demonstrated that LX3 shifted essential amino acid (EAA) content closer toward the “optimal” honey bee diet EAA ratio (as established by DeGroot et al. [42]) compared to 16 common pollen sources (Supplementary Fig. 3B). Principal component analysis (PCA; Supplementary Fig. 3C–F) further revealed EAA profiles of the LX3-containing patty were highly similar to known compositions of beebread—similarities which could relate to the fact that *Api. kunkeei* (present in LX3) is abundantly found in high-quality beebreads [43] and plays a key role in facilitating spontaneous pollen-to-beebread fermentation under natural conditions [44]. Altogether, LX3 fermentation improved nutrient content of the base patty supplement which likely contributed to distinct colony-level effects in the P + LX3 group (Fig. 1). While these findings have major implications related to supplemental feeding practices in beekeeping, non-nutritive factors of LX3 cannot be discounted on the basis that S + LX3 (containing no additional nutrients) also demonstrated colony growth-promoting effects (Fig. 1B–D).

### LX3 treatment increases brood populations without trade-offs in ectoparasite burdens

**Fig. 2:**
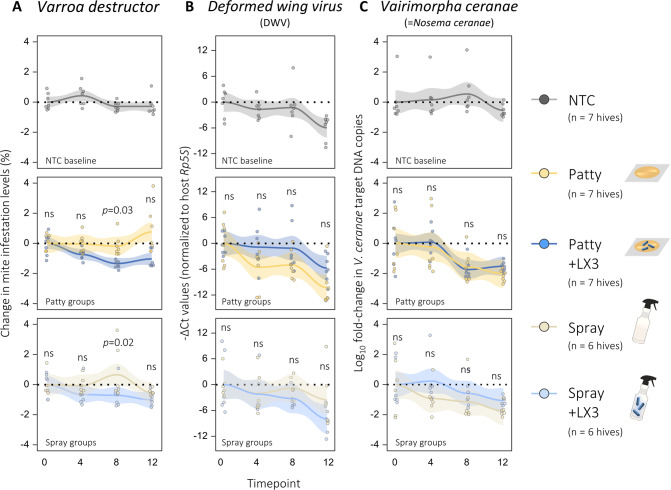
Effect of LX3 treatment against common honey bee parasites. **A** *Varroa destructor* mite populations were measured via standard alcohol wash method. Each data point represents the estimated change in mite population (i.e., number of mites per 100 bees) for an individual hive, with approximately *n* = 300 adult bees sampled per hive (*n* = 33 hives total) at each timepoint. Patty and spray group comparisons shown for three-way ANOVA tests with BH-adjusted *p* values. **B** Viral burden of Deformed wing virus (DWV) and (**C**) microsporidian burden of *Vairimorpha ceranae* were assessed via qPCR-based quantification using RNA and DNA extracted from nurse bee samples, respectively. Each data point represents a distinct hive with three pooled samples taken from each of the 33 hives per timepoint (*n* = 396 total). Patty and spray group comparisons shown for three-way ANOVA tests with BH-adjusted *p* values. Trend lines with 95% confidence bands computed via “loess” method using ggplot2 package in R. ns not significant.

Since total amount of capped brood in a hive can pose safety concerns relating to *Varroa destructor* mite burdens [45], we investigated the impact of LX3 treatment on infestation levels. Baseline levels of *V. destructor* were less than 5% (i.e., 5 mites per 100 adult bees) and similar between all groups at W0, with LX3 treatment (*F*_1,8_ = 11.132, *p* = 0.001) but not timepoint (*F*_1,3_ = 0.914, *p* = 0.438) or vehicle type (*F*_1,8_ = 0.099, *p* = 0.754, three-way ANOVA) affecting these levels over time (Fig. 2A). Pairwise comparisons demonstrated reduced mite burdens at W8 for P + LX3 (*p* = 0.03) and S + LX3 (*p* = 0.02) groups relative to vehicle controls. *Deformed wing virus* (DWV) and *Vairimorpha ceranae* (formerly *Nosema ceranae*) were also assessed given their co-prevenance with *V. destructor* [46], although no vehicle or LX3-specific effects were observable (Fig. 2B, C). These findings support that LX3 treatment can increase brood populations without worsening existing parasite burdens, and furthermore are consistent with a recent 2-year study from Argentina demonstrating certain lactobacilli can exert anti-*V. destructor* properties [47].

### LX3 strains are transiently detectable but do not colonize

Adult “nurse”-aged bees play critical roles in horizontal transfer of gut microbes through trophallaxis, larval rearing activities, and pollen processing [48], and thus provide a well-balanced representation of overall microbial diversity in a hive. To assess in-hive dispersal of the LX3 strains, we performed qPCR-based quantification of *L. plantarum*, *L. rhamnosus*, and *Api. kunkeei* in nurse bees at pre-treatment (W0) and various timepoints post-treatment (Supplementary Fig. 4). Hindgut samples from both P + LX3 and S + LX3 groups at W4 (directly after treatment) demonstrated a detectable increase in *L. plantarum* and *L. rhamnosus* (Supplementary Fig. 4A, B). The S + LX3 group also showed an increase in *Api. kunkeei*, whereas a trend toward increased levels was observed in the P + LX3 group (*p* = 0.1273; Supplementary Fig. 4C). By W24 (20 weeks post-treatment), all levels returned to baseline (Supplementary Fig. 4A–C). These results support the safety of LX3 supplementation, indicate both delivery methods achieve similar dispersal rates, and highlight long-term colonization is not required for LX3 supplementation to be beneficial.

### Multivariate analysis reveals distinct and long-lasting effects of LX3 treatment on nurse bee microbiota

**Fig. 3:**
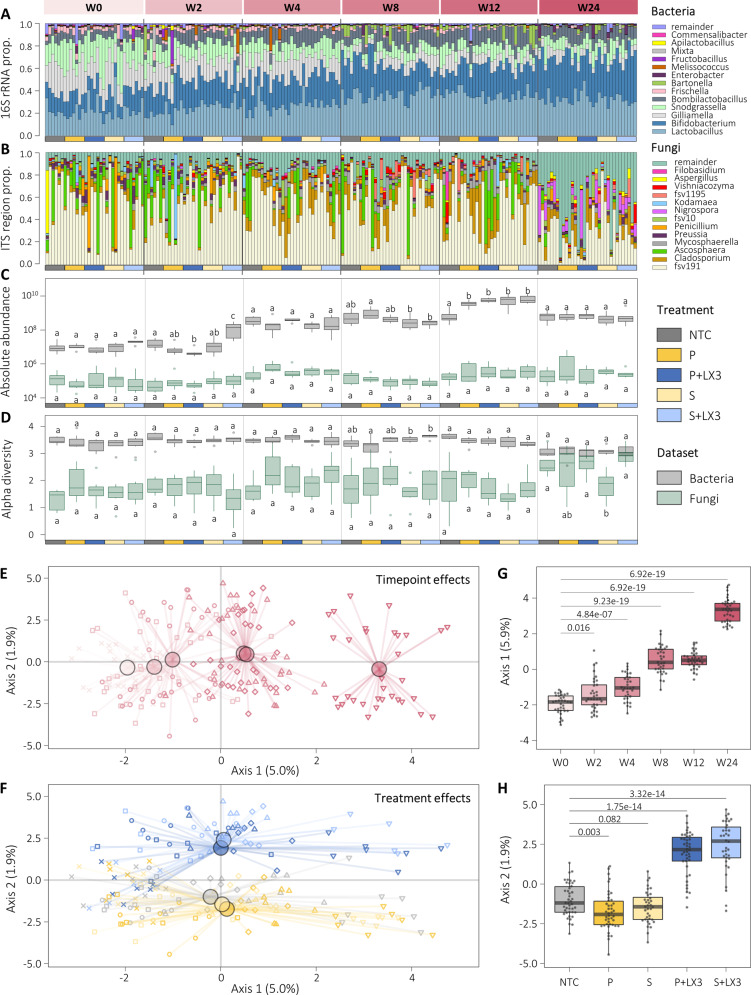
Seasonal trends and LX3 treatment show distinct effects on hindgut microbiota composition. Bar plots shown illustrate the genus-level composition of bacteria (**A**) and fungi (**B**) in nurse bee hindgut samples, as determined via 16S RNA gene and ITS region amplicon sequencing, respectively. Each bar represents a distinct hive with three pooled samples taken from each of the 33 hives per timepoint (*n* = 594 total). **C** Absolute abundance (determined via qPCR-based quantification of the total number of 16S rRNA and ITS copies of DNA) and (**D**) alpha diversity (measured via Shannon’s H Index) of bacterial (gray) and fungal (green) communities in nurse bee hindgut samples. Data depicts the median (line in box), IQR (box), and minimum/maximum (whiskers) values for each treatment group at the specified timepoints. Comparisons shown between treatment groups at each timepoint with statistics derived from two-way ANOVA with BH-adjusted multiple comparisons. **E**, **F** Constrained ordination plots (determined via Aitchison distance-based redundancy analysis; db-RDA) illustrate that microbiota differences are influenced by both timepoint- and treatment-specific effects. **G**, **H** Boxplots show respective timepoint comparisons along Axis-1 (explaining 5.0% of variance) and treatment group comparisons along Axis-2 (explaining 1.9% of variance). Each data point is representative of a distinct hive at a distinct timepoint. Statistics shown for pairwise Wilcoxon tests with BH-adjusted *p* values.

Both 16S rRNA and ITS amplicon sequencing were performed to elucidate the potential impacts of LX3 treatment on indigenous microbial communities found in association with honey bees (Fig. 3). From a sample size of *N* = 594 nurse bee hindguts (randomly and evenly collected from the brood chamber of each of the 33 hives at W0, W2, W4, W8, W12, and W24), we detected a total of 848 bacterial and 3387 fungal unique amplicon sequence variants (ASVs), respectively (Supplementary Data 1C). Genus-level agglomeration of ASVs demonstrated that *Bombilactobacillus* (previously known as *Lactobacillus* Firm-4 phylotype [49]), *Lactobacillus* (Firm-5 phylotype), *Bifidobacterium*, *Gilliamella*, *Snodgrassella*, *Frischella*, *Bartonella*, and *Commensalibacter* were the most dominant bacterial genera (Fig. 3A). Fungal communities showed greater compositional variability with “fSV-g191” (a predicted genus formed by unclassified taxa within the family Myxotrichaceae), *Ascosphaera* (consisting of a single species, *A. apis*—causal agent of chalkbrood), and *Cladosporium* representing the dominant genera found in most samples (Fig. 3B).

Aitchison distance (β-diversity metric) comparisons via permutational multivariate analysis of variance (PERMANOVA) tests identified that timepoint (*F*_5,186_ = 4.52, *p* = 0.001) and LX3 treatment (*F*_1,186_ = 4.13, *p* = 0.001) had significant main effects on microbiota composition, while Wd*-tests (robust to heteroscedasticity [34]) further supported an interaction between these explanatory variables (Wd*_5,187_ = 2.70, *p* = 0.001; Supplementary Data 1I, J). Similar findings were observed using several other distance metrics including Bray-Curtis, Jensen-Shannon, UniFrac, and PhILR (Supplementary Figs. 5 and 6). To visualize the independent effects of LX3 treatment after adjusting for timepoint, constrained ordination was performed via distance-based redundancy analysis (db-RDA; Fig. 3E, F). The centroids of each experimental group clustered around the NTC group on Axis-1 driven by temporal factors (near zero, indicating a good model fit for corrections with *F*_2,196_ = 7.2436 and *p* < 0.0001 for “anova.cca” permutation tests), whereas there was significant deviation along Axis-2 driven by treatment factors as seen for P + LX3 and S + LX3 relative to the vehicle control groups (Fig. 3G, H). A clear separation in microbiota trajectories persisted in both groups at W24 (Fig. 3F), with change point analysis (via a spline-based interpolation method [50]) revealing differences in immediacy of response between P + LX3 and S + LX3 treatments (~3.5 weeks vs. ~1.4 weeks, respectively; Supplementary Figs. 7E, F and 8). Overall, LX3 treatment had long-lasting effects on microbiota composition and delivery method impacted the rate at which these microbial changes occurred.

### Microbial richness and abundance respond to LX3 treatment and correlate with colony size

Since amplicon sequencing does not inform on total microbial abundance [31], we performed qPCR experiments to quantify absolute bacterial and fungal loads. An approximate 100-fold increase in hindgut bacterial loads was observed between W0 and W24 for all treatment groups, whereas fungal loads increased by a lesser degree (<10-fold) over the same period (Fig. 3C). Head samples (including mouthparts) showed identical trends indicating systemic microbial density changes in the hive (Supplementary Fig. 9). In contrast, bacterial α-diversity decreased (−0.32 ± 0.05, *p* = 1.2e−10), while fungal α-diversity increased (0.88 ± 0.15, *p* = 1.6e−07) in all groups between W0 and W24 according to Shannon’s H index (taking species abundance and evenness into account; Fig. 3D) and several other metrics (Supplementary Data 1K, L).Colony size (Fig. 1D) showed moderate to strong positive correlations with bacterial abundance (*r*_s_ = 0.57, *p* = 2.2e−10), fungal abundance (*r*_s_ = 0.32, *p* = 5.6e−06), and fungal α-diversity (*r*_s_ = 0.35, *p* = 6e−07), and a negative correlation with bacterial α-diversity (*r*_s_ = −0.25, *p* = 4.2e−04; Supplementary Fig. 10). Together, these results depict a scenario whereby as the number of adult bees increase in a colony, so does the total bacterial and fungal loads of individual colony members, but with bacterial communities becoming disproportionately abundant (i.e., greater number of bacterial to fungal cells) and dominated by fewer unique taxa as opposed to the concurrent increase in taxonomic richness seen for fungi (Fig. 3C, D).

All LX3 treatments demonstrated clear timepoint-specific effects on absolute abundance and α-diversity metrics, however, these differences were marginal in comparison to the overall magnitude of seasonal trends observed (Fig. 3C, D). One exception was that S + LX3 showed a marked increase in bacterial loads starting at W2 while seasonal increases in the control groups did not occur until W4 (Fig. 3C). A three-way ANOVA supported the main effects of LX3 treatment on hindgut bacterial loads (*F*_1,22_ = 5.923, *p* = 0.0235) and that vehicle type (i.e., patty vs spray delivery method) interacted with LX3 effects (*F*_1,22_ = 6.033, *p* = 0.0224; Fig. 3C). Given that the administered LX3 strains were detectable at similar levels in both P + LX3 and S + LX3 samples (Supplementary Fig. 4), it is likely that any LX3-derived contributions to total hindgut bacterial loads were negligible and not the responsible factor for the unique differences observed.

### LX3 treatment supports symbiont enrichment while reducing bacterial and fungal pathogen burden

We performed differential abundance tests with MaAsLin2 using a generalized linear mixed model (GLMM) approach [51] to delineate species-level microbiota effects of LX3 treatment after adjusting for randomization stratification factors (e.g., hive identity and apiary location) and covariates affecting community structure (e.g., seasonal effects, brood counts, and colony size; see Supplementary Data 1F for full list of variables).

**Fig. 4:**
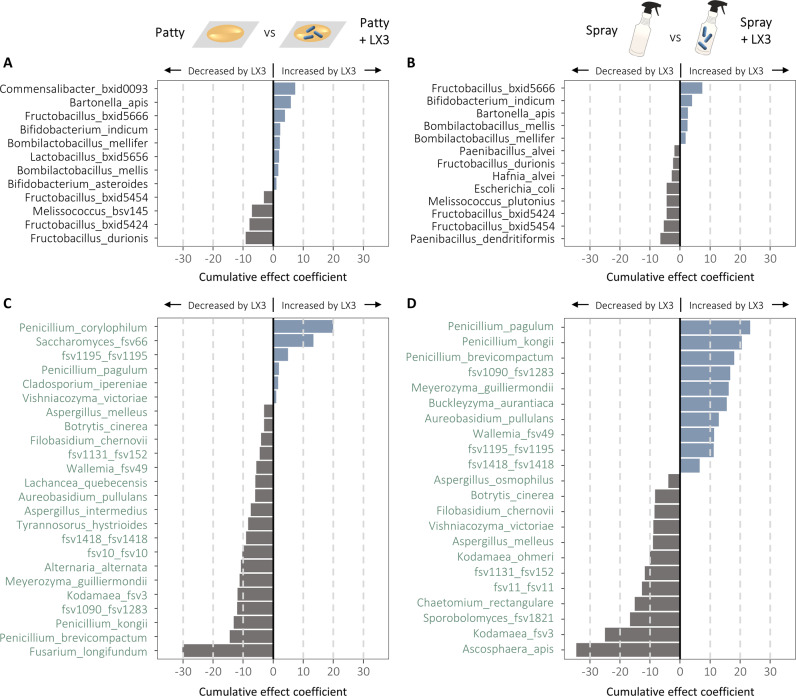
Differentially abundant taxa between LX3 treatment groups and vehicle controls. Differential abundance tests were performed with MaAsLin2 in R using a generalized linear mixed model (GLMM) approach to delineate species-level microbiota effects of LX3 treatment after adjusting for seasonal (timepoint) effects and other covariates (see Supplementary Data 1F). Effect plots shown highlight the top differentially abundant bacteria (**A**, **B**) and fungi (**C**, **D**) for patty and spray group comparisons of interest. Each bar depicts the cumulative effect coefficient of the indicated taxa over the entire 24-week study period (i.e., a sustained difference in taxa abundance was observed following the treatment period). For a comprehensive list including all transient effects and timepoint-specific significant differences, see MaAsLin2 output provided in Supplementary Data 1M, N).

During active supplementation at W2 and W4, LX3 treatment groups consistently showed an enrichment in core bacterial symbionts including *Bifidobacterium*, *Lactobacillus*, *Bombilactobacillus*, and *Bartonella* spp. (Fig. 4A, B and Supplementary Data 1M)—changes resembling those that epitomize thriving colonies [52]. Cumulative effect coefficients across all timepoints demonstrated sustained increases for *B. indicum*, *Bomb. mellis*, *Bomb. mellifer*, and *Bart. apis* in both LX3 groups relative to vehicle controls (Fig. 4A, B). *Fructobacillus bxid5666* also showed sustained increases in both groups, and a pronounced increase in *Commensalibacter bxid0093* was uniquely observed in the P + LX3 group (Fig. 4A). Alongside these changes, several *Melissococcus* and *Fructobacillus* spp. were co-depleted in both LX3 groups, while the S + LX3 group additionally showed a reduction in opportunistic pathogens including *Hafnia alvei*, *Escherichia coli*, *Paenibacillus alvei*, and *Paenibacillus dendritiformis* (Fig. 4B). Thus, LX3 had beneficial impacts on the honey bee microbiota by increasing health-associated symbionts and reducing disease-associated opportunistic pathogens.

Compared to bacteria, fungi showed greater magnitude of differences with distinct response patterns to P + LX3 and S + LX3 treatments (Fig. 4C, D). The P + LX3 group demonstrated the largest number of negative interactions for broad-ranging fungal taxa including *Fusarium longifundum*, *Alternaria alternata*, *Kodamaea* fSV-3, *Aspergillus intermedius*, *Tyrannosorus hystrioides*, *Filobasidium chernovii*, and others (Fig. 4C). However, the most striking overall reduction was for *Ascosphaera apis* in the S + LX3 group, reaching near undetectable levels by W24 (Fig. 4D and Supplementary Data. 1N). Uniquely, the S + LX3 group also demonstrated a cumulative enrichment in *Aureobasidium pullulans*, *Buckleyzyma aurantiaca*, *Meyerozyma guilliermondii* (Fig. 4D)—all of which are expected to be beneficial on the basis of either plant growth-promoting or pathogen-inhibiting properties [53–55]. Another finding of interest was that several antibiotic producing *Penicillium* spp. were increased by S + LX3 treatment, whereas P + LX3 decreased most of them with the exception of *P. corylophilum* and *P. pagulum* (Fig. 4C). These findings highlight that delivery method can greatly affect how LX3 interacts with native fungal communities.

### LX3 modulates expression of host immune factors with microbiota-shaping properties

**Fig. 5:**
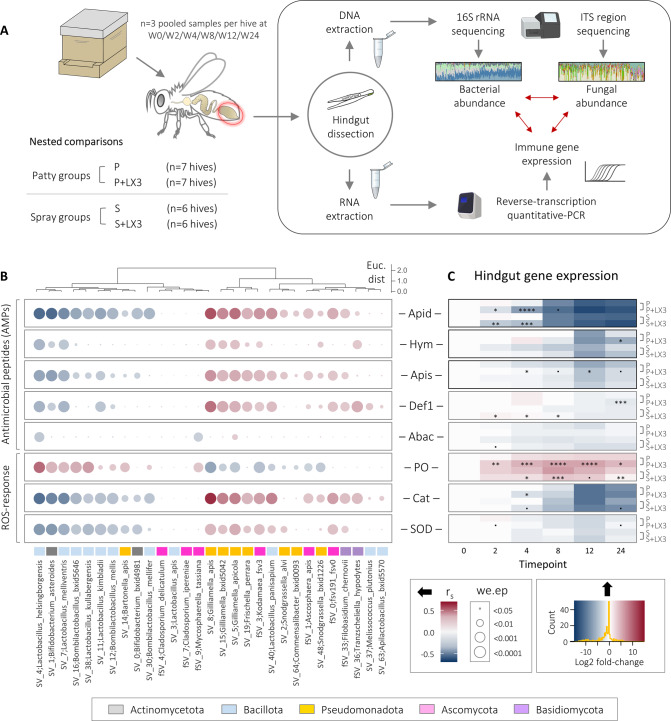
Host immune-microbiota dynamics are influenced by LX3 treatment. **A** Schematic overview of experimental procedure used for obtaining DNA/RNA from matched samples and subsequent analysis of microbial communities and host immune gene expression. **B** Correlation matrix between immune gene expression (determined via qPCR) and species-level microbial abundances in hindgut samples, as determined via the “aldex.corr” function of the ALDEx2 package in R (*r* = Spearman’s Rho, *we.ep* = BH-adjusted *p* values). Hierarchal clustering of samples is shown in dendrogram above and was calculated using the ward.D2 method via the “hclust” function in R. Euc dist = Euclidean distance. **C** Heatmap of immune gene expression with patty and spray group-wise comparisons of interest indicated on the right. Data represent Log2-transformed relative gene expression values (2^−ΔΔCt^ method) normalized to W0 baseline for each group. Statistics shown for LX3 treatment groups (P + LX3 and S + LX3) are based on comparisons to vehicle controls (P and S, respectively) via three-way ANOVA with BH-adjusted multiple comparisons (*n* = 468 total). •*p* < 0.1, *p < 0.05, **p < 0.01, ***p < 0.001, ****p < 0.0001.

Since innate immune signaling and gut microbiota composition are intimately linked in honey bees [12], we postulated that LX3 (possessing established immunomodulatory capacities [20, 27]) could have elicited the observed microbiota changes via host-mediated selective pressures on microbial communities. To assess this possibility, we measured gene expression of five well-characterized antimicrobial peptides (AMPs) and three oxidative response enzymes (Fig. 5 and Supplementary Data 1O).

The expression pattern of *apidacein-1* was unique among AMP genes in showing a progressive downregulation in control groups from W8 onward until W24. Notably, the P + LX3 and S + LX3 groups showed significant downregulation of *apidacein-1* starting at the earlier timepoint of W2, relative to vehicle controls (Fig. 5C). Other important AMP genes such as *hymenoptacein*, *apismin*, *defensin-1*, and *abaecin* exhibited less consistent trends over time, although *hymenoptacein* and *defensin-1* were transiently upregulated in P + LX3 and S + LX3 groups at earlier timepoints (W2 and W4) during the active supplementation period. The latter results are consistent with previous observations from a shorter 4-week supplementation study [20]. Considering oxidative response genes, *catalase* paralleled the expression patterns of *apidaecin* with similar, but less pronounced time- and treatment-dependent downregulation (Fig. 5C). To a lesser extent, *superoxide dismutase* (*SOD*) was also downregulated over time but showed no apparent differences between treatment groups (Fig. 5C). *Phenol-oxidase* (*PO*) expression (traditionally associated with the melanization-immune response and pathogen encapsulation [56]) showed distinct expression patterns compared to all other genes measured and was differentially regulated between LX3 treatment groups at multiple timepoints between W2 and W24 (Fig. 5C). Furthermore, gene expression of dissected heads (proxy for social immunity [57]) were similar hindguts (individual immunity) for both timepoint- and treatment-specific patterns, demonstrating the observed effects were systemic rather than tissue-specific (Supplementary Fig. 7G).

### Host immune response to LX3 treatment is associated with altered microbial network dynamics

To assess how LX3-induced immune effects may have played a role in the observed microbiota shifts (Fig. 3), we used NetCoMi [58] to construct an interkingdom (bacterial-fungal) co-occurrence network (Supplementary Fig. 7A–D and Supplementary Data 1P) and then analyzed correlations with hindgut tissue gene expression using a dual RNA/DNA extraction approach (Fig. 5). Findings elucidated distinct clustering patterns that largely corresponded with phylogenetic relatedness at the phylum-level, although several exceptions were apparent. *Bacillota* and *Actinomycetota* clustered together including the bona fide symbionts *Bombilactobacillus*, *Lactobacillus* (exclusively Firm-5 phylotypes), and *Bifidobacterium* spp., all of which were associated with reduced gene expression for most AMPs (especially *apidaecin*) but increased expression of *PO* (Fig. 5B)—a pattern consistent with the enrichment of these taxa in LX3 treatment groups (Fig. 4A, B). Nearly identical trends in host immune-microbiota associations were seen for a single *Pseudomonadota* symbiont, *Bartonella apis*, which uniquely clustered with the aforementioned taxa from different phyla (Fig. 5B). Exact opposite immune association patterns were seen for other *Pseudomonadota* members (e.g., *Gilliamella apis*, *Gilliamella apicola*, *Frischella perrara*, and *Snodgrassella alvi*), which supports the in vitro findings that purified apidaecin and hymenoptaecin derived from honey bee gut tissue can selectively inhibit the growth of these bacteria [5]. Environmental opportunists and host-adapted pathogens that were reduced by LX3 treatment (e.g., *M. plutonius*, *A. apis*, *Kodamaea* spp.) also clustered predominantly with *Pseudomonadota* (Fig. 5B). The cumulative findings show that transient immune responses to LX3 treatment differed slightly based on delivery method but were overall associated with long-term beneficial effects on microbiota composition. It is important to highlight that these microbial relationships do not necessarily indicate a causative immune response by the host, but rather support the notion that reciprocal eco-evolutionary dynamics may drive the formation of distinct microbiota configurations in bees.

## Discussion

This study demonstrated a range of health benefits associated with LX3 supplementation and further identified that some of these effects were strictly dependent on the way in which the strains were delivered to the hive. Superior efficacy in reducing fungal pathogens was a notable advantage of the spray formula, whereas the patty formula performed considerably better in terms of increasing capped brood and overall colony growth throughout the summer season. While the spray-specific effects could be attributed to physical contact differences in the hive upon administration, our findings indicate that the patty-specific effects were driven by nutritional enhancements of the base pollen patty ingredients via LX3 fermentation, which is a process that aligns with how bees naturally ferment pollen into beebread for improved digestibility [59]. These findings directly expand on past work showing that patty-based LX3 supplementation can rescue antibiotic-induced brood deficits [20] likely originating from impaired protein digestion related to gut microbiota damage [60, 61]. It should be noted, however, that spray-based delivery of LX3 still increased capped brood and colony size beyond baseline levels observed in the control groups (Fig. 1), thus suggesting that LX3-mediated immune and microbiota effects alone can contribute to beneficial outcomes at the colony-level.

There were no obvious safety concerns identified. Spray and patty delivery methods led to detectable increases of the LX3 strains in honey bee hindguts following treatment at W4, and these levels returned to baseline by W24 (Supplementary Fig. 4). This highlights that both delivery methods were efficacious for their intended purposes of supplementing viable bacteria to the hive and that colonization was not required for host benefits to be derived. Notably, probiotics frequently do not colonize the host and immune stimulation by allochthonous strains is often effective than autochthonous strains in promoting disease resistance [62]. Moreover, while both LX3 treatment groups experienced long-lasting changes in microbiota composition, there was no evidence to support displacement of core gut symbionts or any other lasting negative impacts (Figs. 3 and 4). Beyond evaluating the treatment groups of interest, this study facilitated several discoveries relating to temporal immune-microbiota dynamics and cross-kingdom microbial interactions in the honey bee hindgut.

Hindgut bacterial communities play a crucial role in regulating honey bee immunity [12] and microbiota dysbiosis (often ambiguously defined, but with the simplest criteria being a relative depletion of symbiotic gut bacteria) can increase host susceptibility to a broad range of diseases [63, 64]. Here, we show that in California over the summer months (following almond harvest) there was a seasonal trend toward improved microbiota composition (Fig. 3). This was primarily characterized by an increased absolute abundance of core *Bombilactobacillus*, *Lactobacillus*, and *Bifidobacterium* spp.—all of which are carbohydrate utilization specialists [9, 65, 66] widely associated with healthy colony outcomes [52, 67]. Uniquely, *Bart. apis* (highly prevalent *Alphaproteobacteria* member in honey bees) was also increased alongside these taxa which may be related to syntrophic partnerships as seen in other insect-adapted symbionts [68]. Kešnerová et al. [69] observed highly similar trends repeatedly in a 2-year longitudinal study in Switzerland, which highlights the overall reproducibility of the current findings on a global-scale. Adding to this knowledge, we show there are clear seasonal trends in immune gene expression that closely overlap with microbiota shifts, and that LX3 treatment can modulate immune-microbiota dynamics in a way that supports further enrichment of the aforementioned symbionts (Figs. 4 and 5). While the underlying drivers of this biological phenomenon remain unclear, cumulative cross-continental evidence indicates a mechanism distinct from spatially-dependent environmental factors (at least within temperate climates [70, 71]) known to impact honey bee microbiota composition, such as differences in forage type [72] and pesticide exposure [73].

Compared with bacteria, little is known about the temporal dynamics of fungi in honey bees. Our findings support the notion that most fungi are transient colonizers derived from the environment [74]. However, we also identified clear seasonal trends in fungal taxa and a distinct set of highly prevalent species (found in >80% samples) which suggests the existence of a “core mycobiome” (Supplementary Data 1C). One unclassified species in particular, fSV-0 (predicted member of Myxotrichaceae—a fungal family commonly reported in bees across multi-continental studies [75–78]), warrants immediate investigation on the basis it was found in 100% of samples (generally dominant by abundance) and could conceivably have a major impact on honey bee health. Notably, almost all fungal taxa (including fSV-0) showed a strong negative correlation with *Bombilactobacillus*, *Lactobacillus*, and *Bifidobacterium* spp. (Supplementary Fig. 7A–D). These findings are consistent with reported inhibitory effects of lactic acid- and acetic acid-producing bacteria on diverse fungi [15, 79, 80], which together could also explain the broad anti-fungal effects seen in LX3-supplemented groups (Fig. 4).

An illuminating discovery relating to hive disease dynamics was that spray-based LX3 far outperformed patty-based LX3 in reducing the two major brood pathogens, *M. plutonius* and *A. apis* (Fig. 4). Bacteriocin production of Kunkecin A by honey bee-derived *Api. kunkeei* BR-1 (present in LX3), as has been shown for other *Api. kunkeei* strains [81], is a plausible mechanism supporting activity against *M. plutonius* but requires further validation. In contrast, the intrinsic ability of LX3 strains to produce lactic acid likely explains the strong activity against *A. apis* [80]. Nonetheless, localized spray delivery and coating of brood cells (i.e., where *M. plutonius* and *A. apis* are most abundant) appears to be the most crucial factor influencing overall efficacy of LX3 strains against these pathogens. This point also extends to opportunistic fungi colonizing inner hive surfaces, such as *Kodamaea* spp., which through producing high levels of isopentyl acetate (a major component of honey bees’ alarm pheromone [82]) induce chronic stress and (in the case of *K. ohmeri*) can attract small hive beetle (*Aethina tumida*) and other parasites [83]. Our findings show that *K. ohmeri* as well as an unclassified *Kodamaea* sp. (fSV-3) were dramatically reduced in response to spray-based LX3 (Fig. 4)—an effect worthy of future study given the conceivable benefits on hive-level energetic burden. Moreover, both pollen- and spray-based LX3 approaches demonstrated distinct activities against *Alternaria alternata* (fungal agent of leaf spot in 380 plant species [84]), *Fusarium longifundum* (fungal agent of canker disease in almond crops [85]), and several other opportunistic pathogens (Fig. 4 and Supplementary Data 1M, N) for which honey bees are recognized vectors [74]. Alongside the emerging realization that multi-host epidemics are especially common within plant-pollinator networks [86], these results cumulatively suggest that LX3-mediated inhibition of plant and insect pathogens could greatly benefit wild bees and aid in crop pest management.

Linking the ideas discussed so far with honey bee immunobiology and nutrition, we identified clear phylogenetic clustering of bacterial and fungal taxa based on correlations with host expression of AMPs (innate immune effectors closely linked with microbiota composition [87] as well as protein intake [88]) and oxidative stress-response enzymes (Fig. 5). The strongest signal was for *apidaecin-1* (encoding a proline-rich AMP targeting Gram-negative bacteria), which showed drastic downregulation over time corresponding most evidently with a decline in *Pseudomonadota* as well as select *Bacillota* (e.g., *L. panisapium* and *L. apis*). These results are corroborated by experimental evidence showing selective toxicity of *apidaecin-1* isoforms against most *Pseudomonadota* (including *G. apicola*, *S. alvi*, *F. perrara*, and environmental *Escherichia coli*) [5] and that some (but not all) *L. apis* strains possess unique S-layer proteins that activate Toll signaling-mediated *apidaecin-1* expression [89]. A noteworthy point to highlight is that LX3 supplementation accelerated the seasonal downregulation of *apidaecin-1* (in hindguts and also hypopharyngeal tissue in head samples, demonstrating a systemic effect) in a delivery-*independent* manner (i.e., nutrient factors likely not involved), while also transiently upregulating *apisimin*, *hymenoptacein*, and *defensin-1* involved in bacterial and fungal pathogen resistance (Fig. 5). The latter effects are highly consistent with past work [20] demonstrating LX3 can broadly upregulate insect AMPs (via peptidoglycan recognition protein-mediated activation of the IMD pathway), whereas the contrary findings for *apidaecin-1* may be indirectly related to destabilized microbial networks stemming from antibiotic exposure in the previous study.

In terms of non-AMP immune genes, *phenol-oxidase* showed a strong seasonal upregulation in hindguts (but not head samples; Supplementary Fig. 7) and could potentially be related to changes in fungal-derived β-glucan which are known to cause a dose-dependent increase in *phenol-oxidase* expression [90]—an effect also associated with improved survival to DWV [91]. On that note, no major differences in DWV-immune dynamics were found in the current study (Fig. 2B). Although, relatedly, *Varroa* mite infestation rates showed subtle decreases following LX3 treatment with 7/7 hives responding to patty delivery and 5/6 hives responding spray delivery (Fig. 2A). Similar findings were observed during a recent 2-year study in Argentina showing a 50–80% reduction in mite loads via *Lactobacillus salivarius* A3iob supplementation [47], however, the mechanism for such effects remains elusive. Connecting the insect gut-brain axis [92], neuro-immune interactions [93], and how beneficial bacteria influence hygienic behaviors represent promising future directions.

In summary, this study has: (1) introduced a novel spray-based delivery method for supplementing beneficial lactobacilli to honey bees, (2) demonstrated that LX3-induced immune and microbiota effects can influence multiple pathogen loads, brood development, and overall colony size, and (3) advanced our understanding of seasonal microbiota variation with regard to bacterial-fungal interactions in the hindgut over time. Given the non-overlapping benefits associated with patty- and spray-based LX3 treatments, a combined approach is advisable in future studies. Altogether, the findings emphasize the importance of considering delivery method in probiotic-focused disease management strategies. Application of these approaches to address unsustainable colony loss in the beekeeping industry warrants urgent consideration.

## Supplementary information

Supplementary Information

Supplementary Dataset 1

## Data Availability

All underlying data from the figures in this study have been made available in a multi-tabbed excel document with self-contained legends describing each of the tabs (Supplementary Data 1). Sequencing data for 16S rRNA and ITS datasets have been uploaded to the NCBI Sequence Read Archive (SRA) database under accession identifier PRJNA856263 and PRJNA856341, respectively. Code and command line input settings used for data interpretation, statistical analysis, and figure generation is available at https://github.com/bdaisley/LX3CA1.
