# Supplementary material for: Delivery mechanism can enhance probiotic activity against honey bee pathogens
Source: ISME J. 2023 Jun 14;17(9):1382–95. doi: 10.1038/s41396-023-01422-z (PMC10432525; doi:10.1038/s41396-023-01422-z)
Supplement: Supplementary file 1 — Supplementary Information [file 41396_2023_1422_MOESM1_ESM.pdf]

Supplementary Information

Delivery mechanism can enhance probiotic activity against honey bee pathogens

Daisley et al.

This PDF file includes:

Supplementary Figures 1-10

Other Supplementary Materials for this manuscript include the following:

Supplementary Dataset 1

SUPPLEMENTARY FIGURE 1

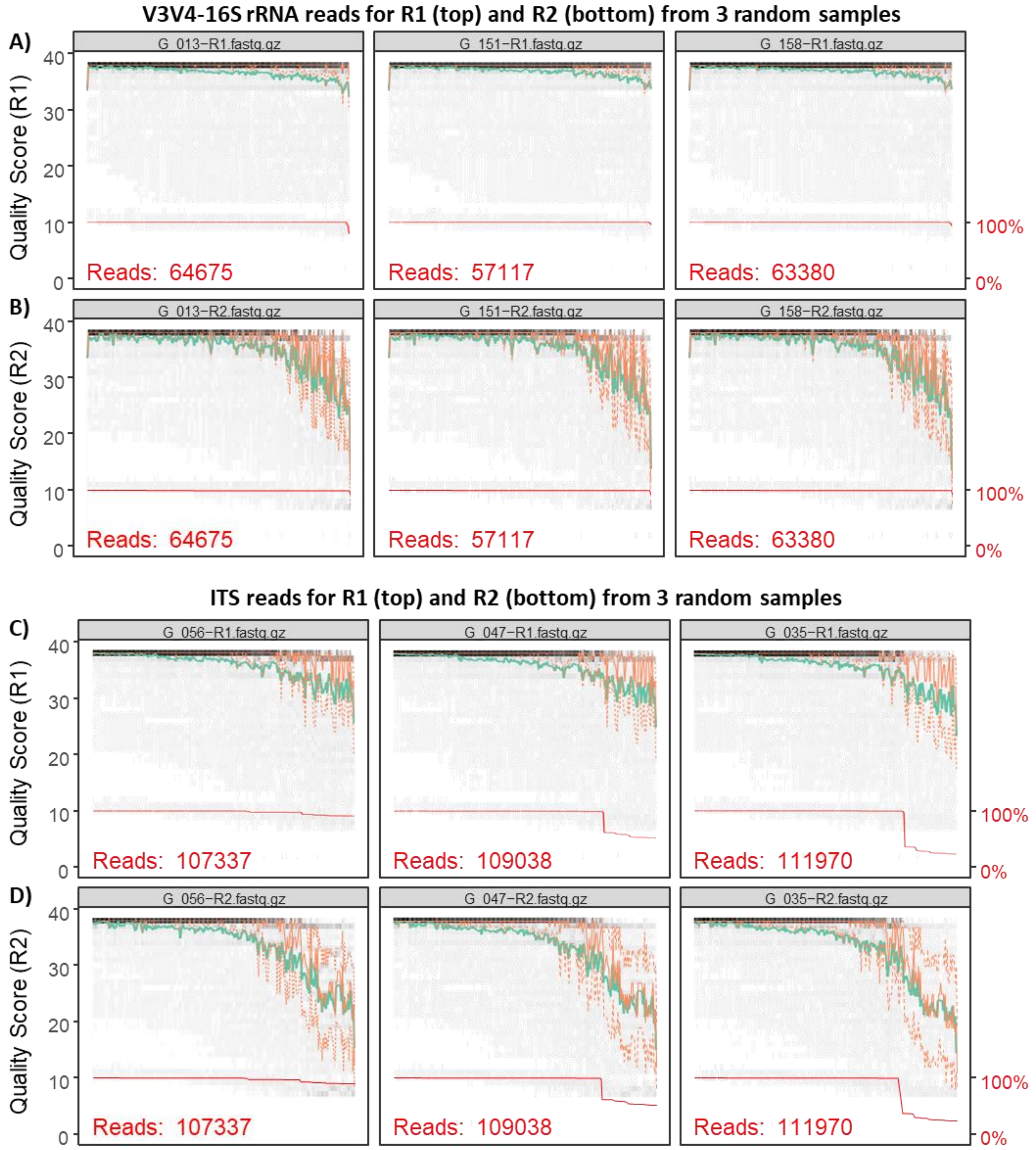

Supplementary Figure 1. Quality profiles for sequencing data. Visualization of quality profiles for forward and reverse reads from the **(A-B)** 16S rRNA gene and **(C-D)** ITS region sequencing datasets. Plots generated in R using the ‘plotQualityProfile’ function of DADA2 for three random samples per dataset. The black underlying heatmap depicts the frequency of each score at each base position. Green solid lines = mean quality scores, orange solid lines = median quality scores, orange dotted lines = quartiles for quality scores.

SUPPLEMENTARY FIGURE 2

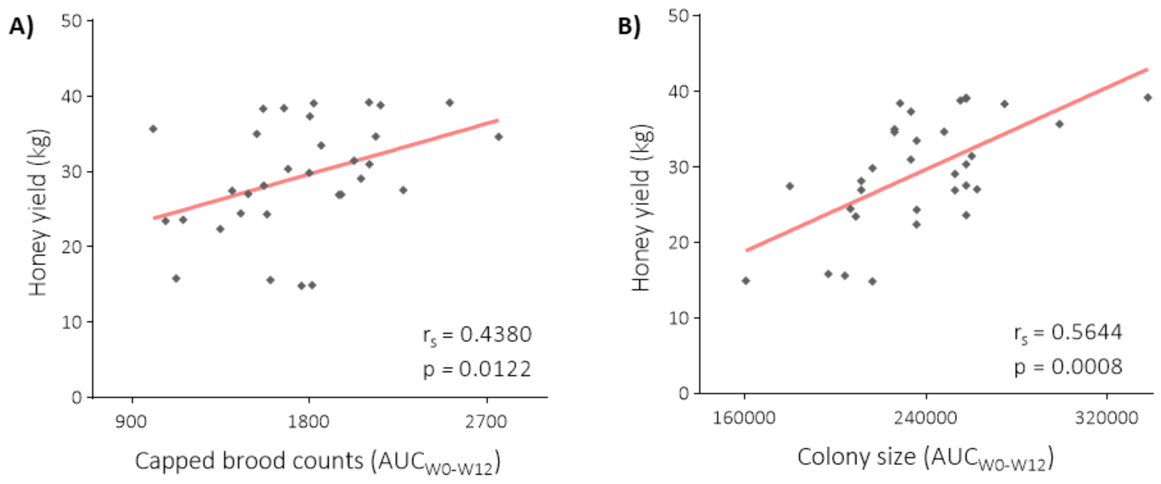

Supplementary Figure 2. Correlations between honey yield and hive population metrics. Scatterplots for honey yield (measured at W12) compared to area under curve (AUC) measurements between W0-W12 for **(A)** capped brood counts and **(B)** colony size. Spearman correlation statistics shown for each comparison.

SUPPLEMENTARY FIGURE 3

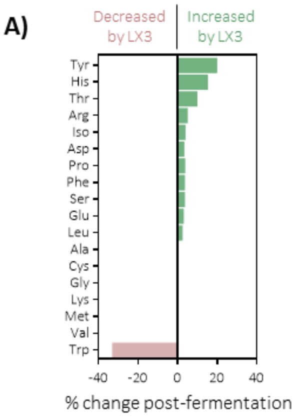

B)

Essential amino acids (g/16g N)									
Arg	His	Ile	Leu	Lys	Met	Phe	Thr	Val	Source
3.0	1.5	4.0	4.5	3.0	1.5	2.5	3.0	4.0	(1) Optimal
6.1	2.5	3.3	5.6	4.8	1.3	3.3	3.0	4.1	(2) Beebread
5.8	2.1	3.6	5.7	4.3	1.0	3.9	3.1	3.8	(3) LX3-patty
4.7	2.2	4.4	8.0	3.3	2.1	4.2	4.4	5.4	(4) Lupin
4.7	1.9	4.8	6.8	5.6	1.6	3.8	5.1	5.9	(5) Maize
4.7	2.5	4.4	7.0	5.9	2.2	4.3	4.6	5.3	(6) White clover
3.7	4.6	4.3	6.6	5.8	2.2	3.7	4.0	4.6	(7) Sunflower
5.1	2.1	4.8	6.7	6.2	2.2	4.2	4.6	5.2	(8) Faba bean
5.5	1.9	4.3	6.4	6.5	2.6	3.9	4.5	5.1	(9) Almond
4.8	2.6	4.1	6.9	6.4	2.4	4.2	4.4	5.4	(10) Pear
4.3	2.7	4.5	6.8	6.9	2.0	3.9	4.2	5.0	(11) Buckwheat
4.3	3.7	3.6	6.0	6.4	2.2	4.1	4.2	4.5	(12) Lavender
4.5	4.4	5.0	6.9	6.8	2.6	4.2	4.1	5.7	(13) Saffron thistle
6.2	3.0	4.0	7.2	6.9	1.1	4.4	3.5	4.6	(14) Cranberry
4.8	2.7	4.5	7.6	8.5	2.3	4.0	5.1	5.6	(15) Canola
6.8	3.9	4.5	7.5	7.1	1.8	4.5	3.8	5.1	(16) Blueberry
5.2	2.3	4.2	7.2	9.5	2.1	4.3	4.4	5.3	(17) Citrus
2.4	6.5	2.5	2.5	8.3	1.4	2.8	3.0	1.1	(18) Dandelion

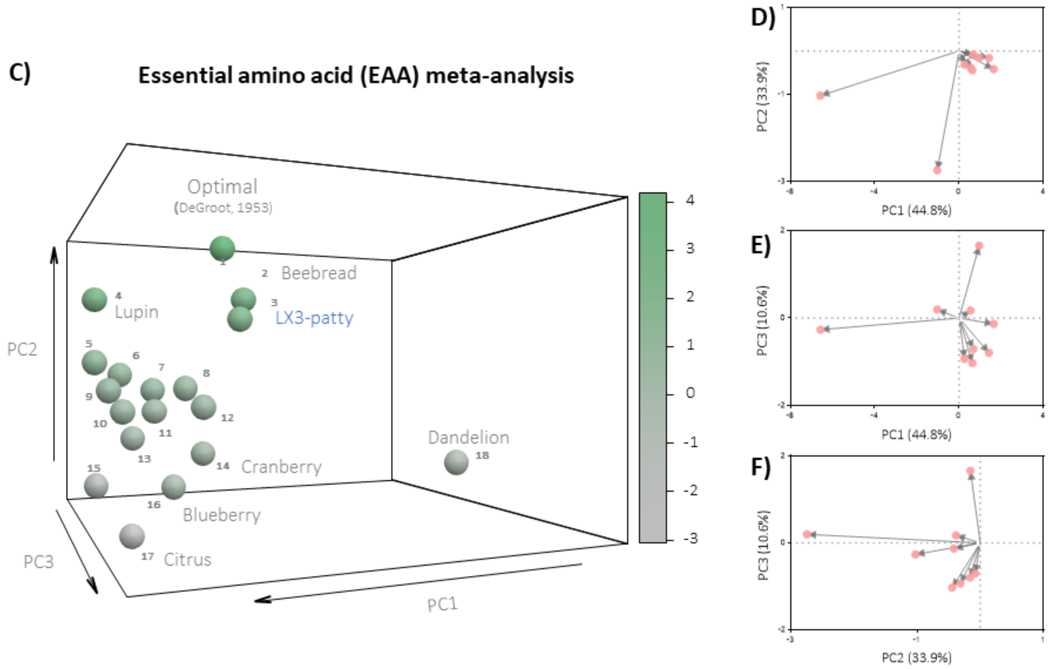

Supplementary Figure 3. LX3-fermentation improves nutritional profile of pollen patty delivery vehicle. (A) LX3-induced changes in amino content of pollen patty following 7-day incubation. **(B)** Summary table of EAA in the P+LX3 patty (this study), beebread collected from a farmland apiary[1], and 16 common pollen sources from past literature[2] including lupin (*Lupinus augustifolius*), maize (*Zea mays*), white clover (*Trifolium repens*), sunflower (*Helianthus annuus*), faba bean (*Vicia faba*), almond (*Prunus dulcis*), pear (*Pyrus communis*), buckwheat (*Fagopyrum esculentum*), lavender (*Lavandula* spp.), saffron thistle (*Carthamus lanatus*), cranberry (*Vaccinium angustifolium*), canola (*Brassica napus*), blueberry (*Vaccinium macrocarpon*), citrus (*Citrus* spp.), and dandelion (*Taraxacum officinale*) crops. **(C)** Principal component analysis (PCA) plot exploring EAA content from the listed sources. Points depicts PC scores for sources 1-18, with the distance between each point representing differences in amino acid composition. Points closer to "Optimal" are more balanced than points further away based on the physiological demands of honey bees[3]. Colour scale to the right represents the PC2 axis and is shown for purposes of depth perception. **(D-F)** Loadings plots for PC1-PC3 showing the directional pull of amino acids (red points) on each axis. Arrow lengths are proportional to the strength of directional association for each amino acid.

SUPPLEMENTARY FIGURE 4

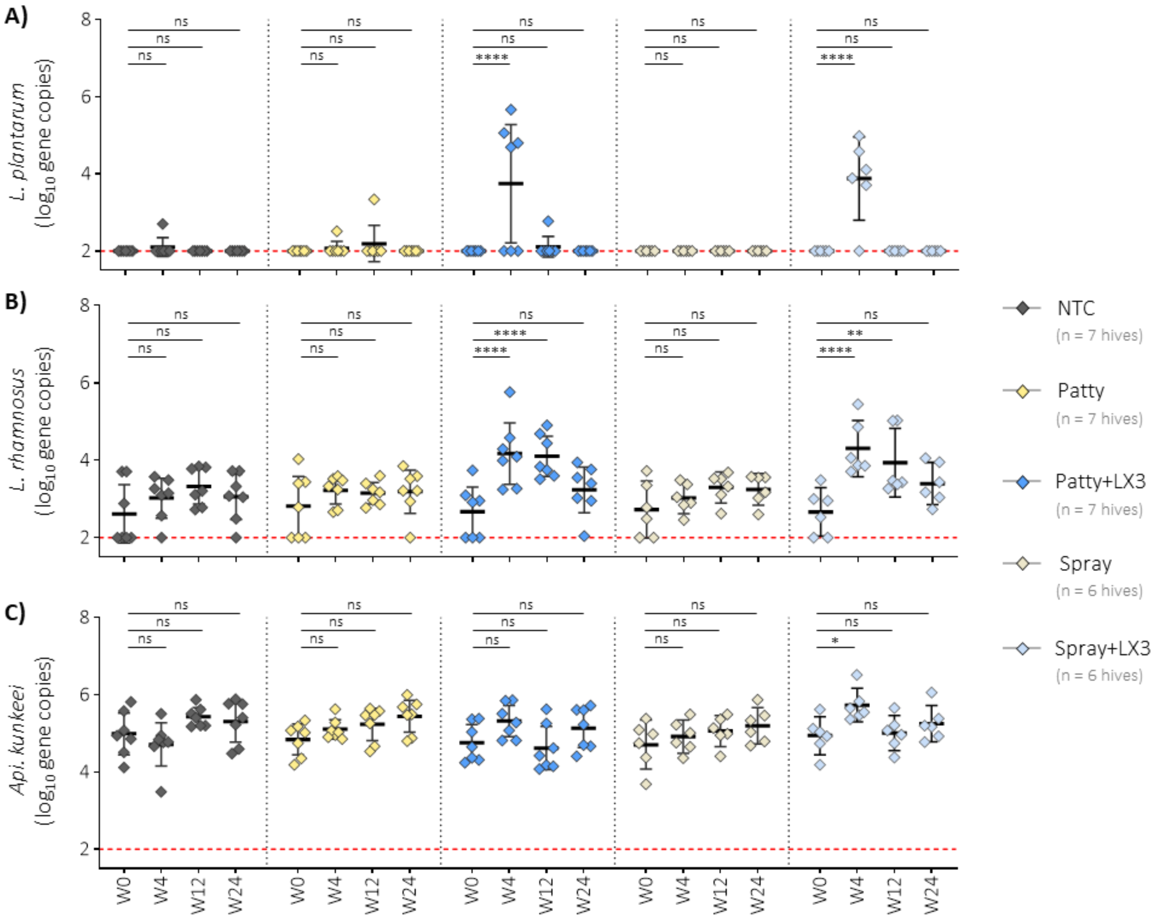

Supplementary Figure 4. LX3 strains are transiently detectable in nurse bee samples but do not colonize. Species-level quantification of **(A)** L. *plantarum*, **(B)** *L. rhamnosus*, and **(C)** *Api. kunkeei* was performed via qPCR on nurse bee samples before treatment W0, directly after treatment (W4), 8-weeks post-treatment (W12), and at the final timepoint 20 weeks post-treatment (W24). Data depicts the mean ± 95% confidence intervals. Statistics shown for two-way ANOVA with Tukey's multiple comparisons. *p<0.05, ***p<0.001 and ****p<0.0001, ns = not significant.

SUPPLEMENTARY FIGURE 5

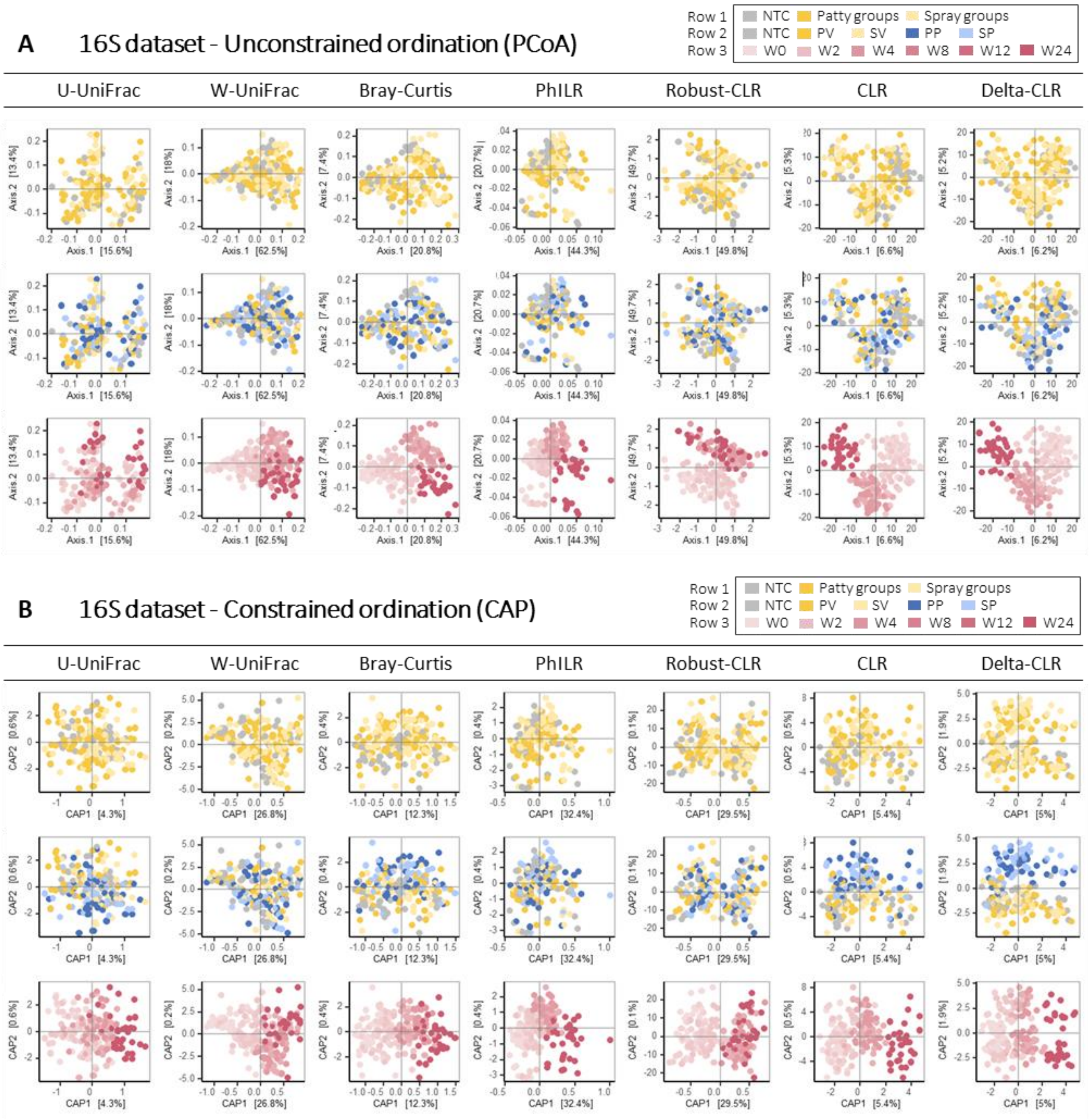

Supplementary Figure 5. Beta diversity ordination plots for 16S rRNA gene sequencing data. Unconstrained (A) and constrained (B) ordination plots as determined by principal coordinate analysis (PCoA) and distance-based redundancy analysis (db-RDA). Unweighted-UniFrac (U-UniFrac), weighted-UniFrac (W-UniFrac), Bray-Curtis, and CLR-based ordinations were calculated using the ‘phyloseq’ package (v1.36.0) in R. PhiLR ordination was calculated using the ‘philr’ package (v1.18.0) in R. Robust CLR (rCLR) ordination calculated using the ‘deicode’ plugin for QIIME2 (v2021.4).

SUPPLEMENTARY FIGURE 6

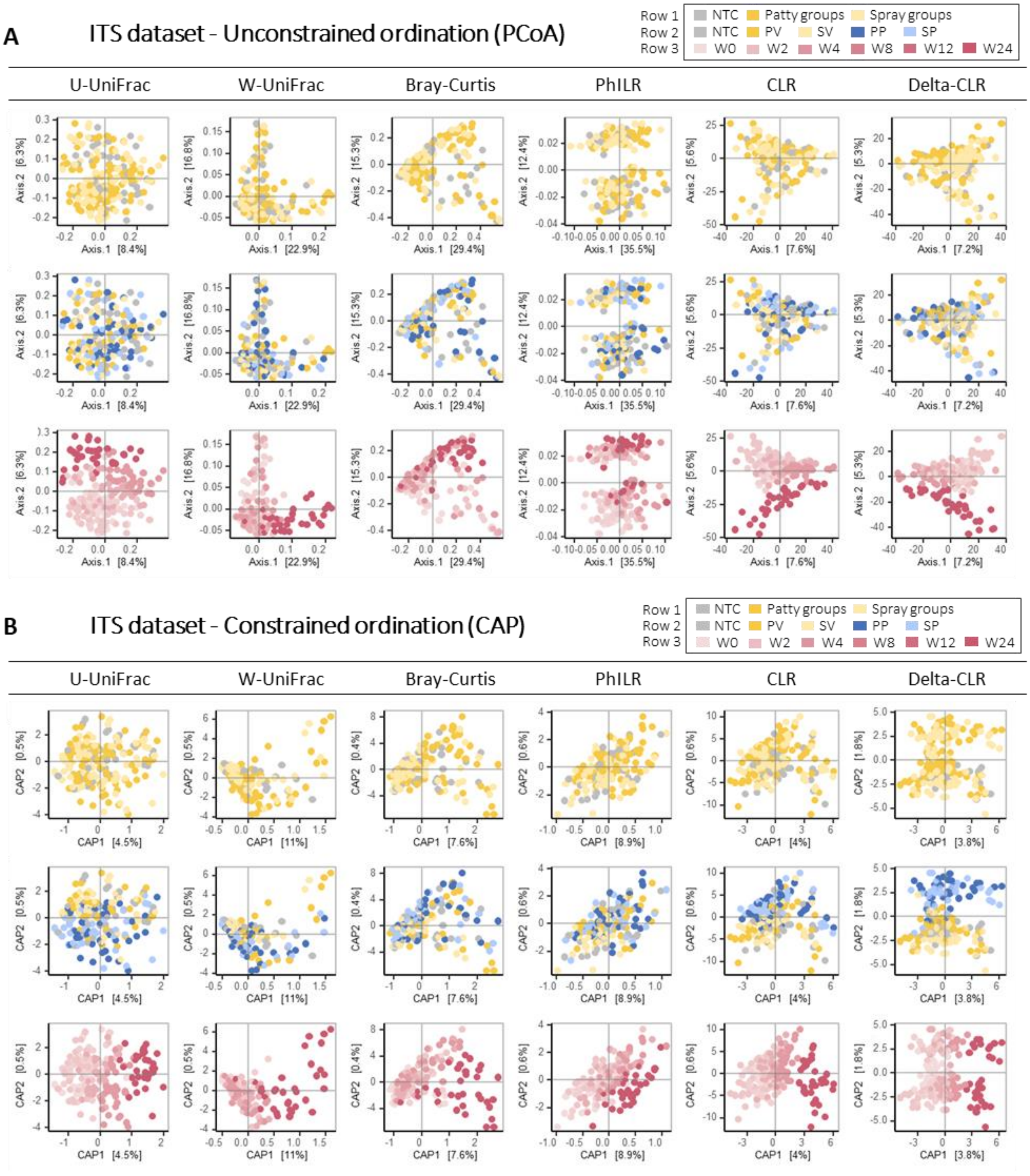

Supplementary Figure 6. Beta diversity ordination plots for ITS region gene sequencing data. Unconstrained (A) and constrained (B) ordination plots as determined by principal coordinate analysis (PCoA) and distance-based redundancy analysis (db-RDA). Unweighted-UniFrac (U-UniFrac), weighted-UniFrac (W-UniFrac), Bray-Curtis, and CLR-based ordinations were calculated using the ‘phyloseq’ package (v1.36.0) in R. PhiLR ordination was calculated using the ‘philr’ package (v1.18.0) in R.

SUPPLEMENTARY FIGURE 7

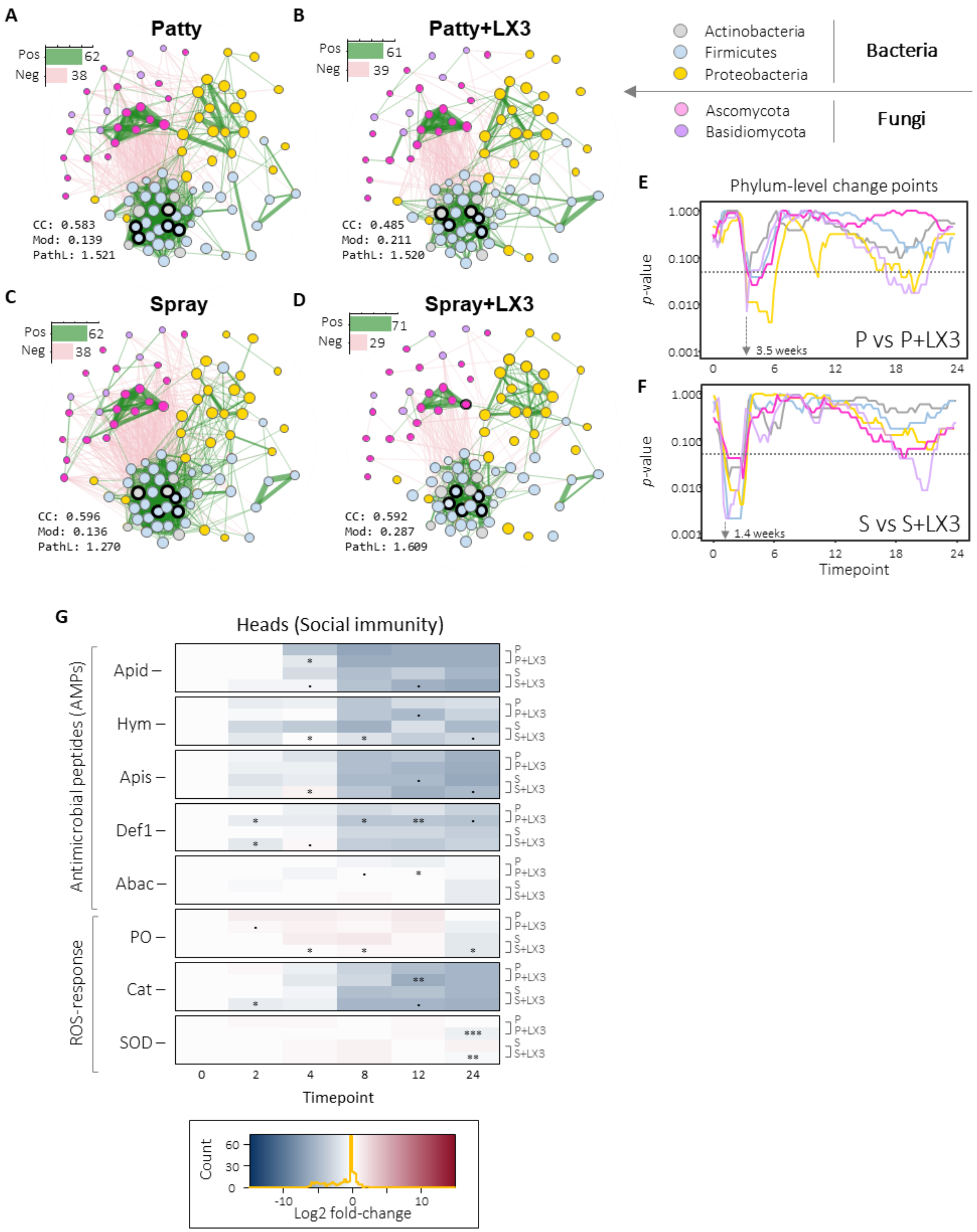

Supplementary Figure 7. Microbial co-occurrence networks of bacterial and fungal communities. (A-D) Microbial co-occurrence network graphs visually illustrate the significant correlations (edges; Spearman's Rho, $r_s >= |0.3|$ and BH-adjusted $p < .05$) between the absolute abundance of bacterial and fungal taxa (nodes) in honey bee hindgut samples. Statistics determined via the NetCoMi package in R using all-timepoint sample data as input for P vs P+LX3 and S vs S+LX3 group comparisons. CC= Clustering coefficient, +E= % positive edges, Mod=Modularity, PathL=Path length. **(E-F)** Sliding window plots for patty and spray group comparisons highlight the distribution of timepoint-specific significant differences in phyla abundances between the specified groups. Statistical analysis including spline-based interpolation of gaps in time series data was performed using the splinectomeR package in R. **(G)** Immune gene expression of head tissue samples. Group-wise comparisons of interest are indicated on the right. Data represents Log2-transformed relative gene expression values (2-ΔΔCt method) normalized to W0 baseline for each group. Data derived from 26 distinct hives (n=7/7/6/6 for P/P+LX3/S/S+LX3 groups, respectively) with from a total of n=126/126/108/108 individuals (sampled evenly from hives, respectively) assessed across the six timepoints shown. Statistics shown for LX3 treatment groups (P+LX3 and S+LX3) are based on comparisons to the vehicle controls (P and S, respectively) via three-way ANOVA with BH-adjusted multiple comparisons. •p<0.1, *p<0.05, **p<0.01. Heatmaps were generated via the heatmap.2 function of the gplots package (v3.1.1) in R.

SUPPLEMENTARY FIGURE 8

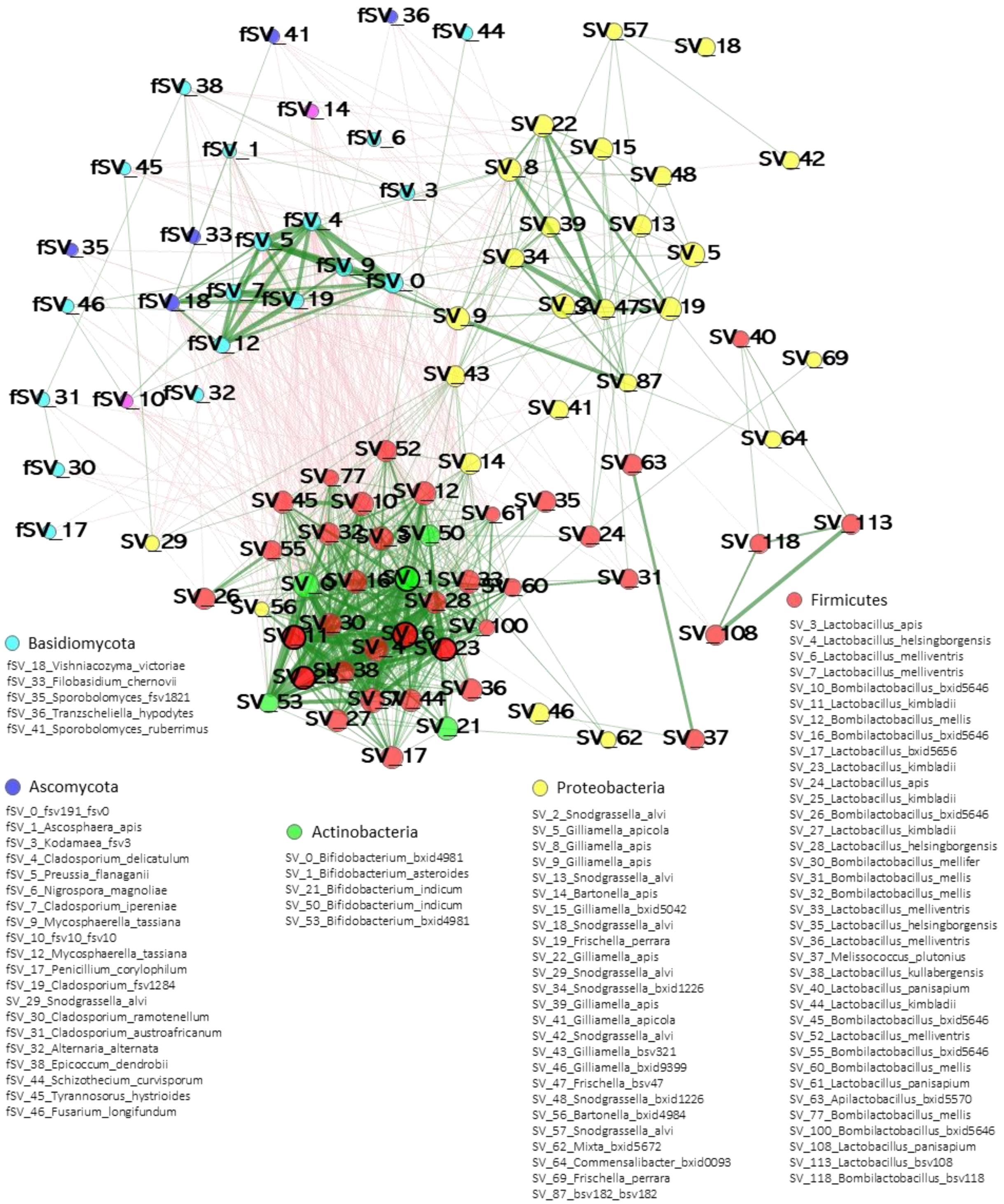

Supplementary Figure 8. Enlarged microbial co-occurrence network with labels. The network shown was constructed using the NetCoMi package in R and represents an enlarged replica of the patty vehicle network in Supp. Fig. 7A. Related statistically analyses and descriptive network properties are available in Supp. Data 1P.

SUPPLEMENTARY FIGURE 9

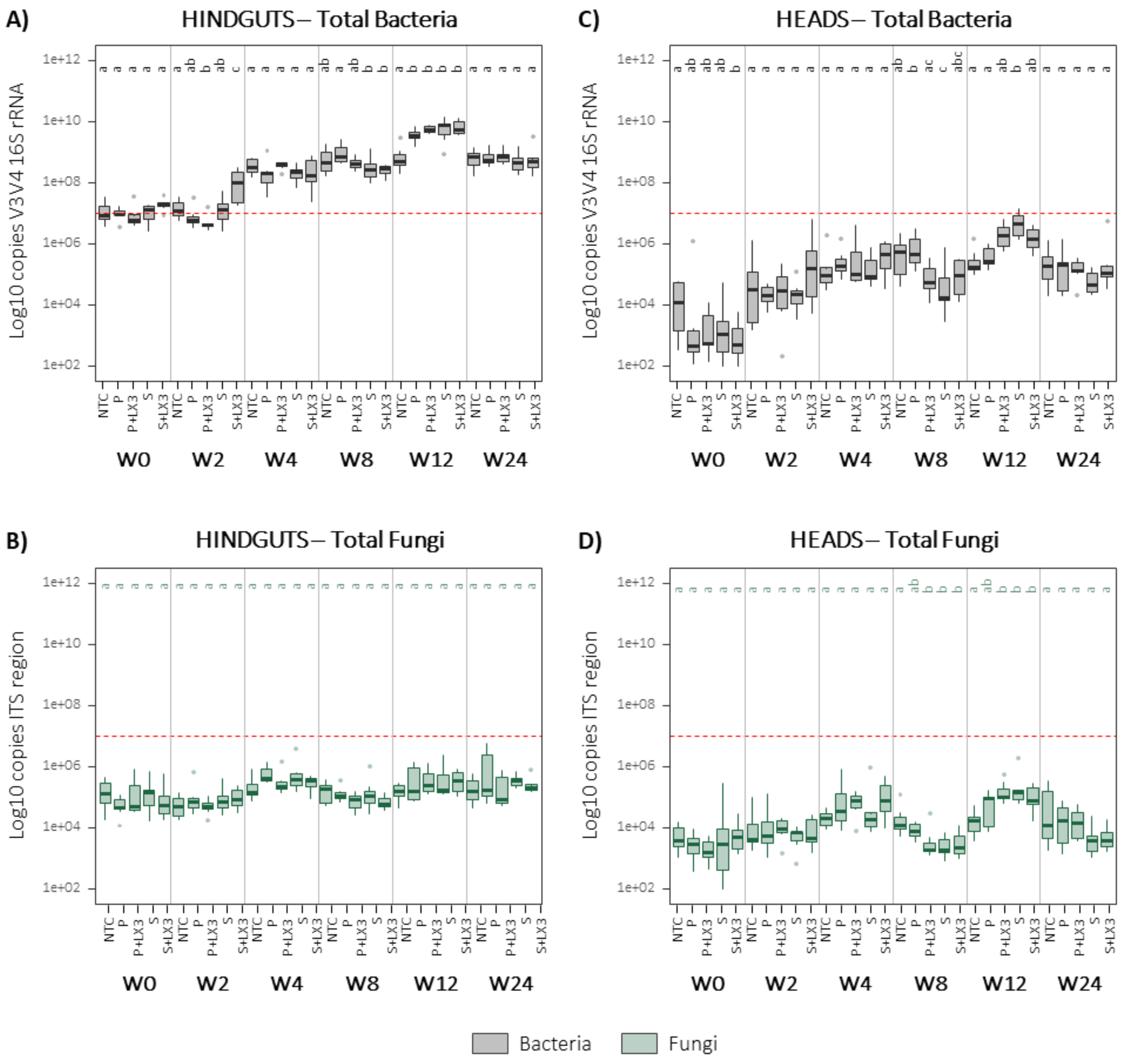

Supplementary Figure 9. Bacterial and fungal loads differences between hindgut and head samples. Absolute abundances were estimated via qPCR using bacterial (Bakt_341F: 5’-CCTACGGGNGGCWGCAG-3’, Bakt_805R: 5’-GACTACHVGGGTATCTAATCC-3’) and fungal (ITS1f: 5’-CTTGGTCATTTAGAGGAAGTAA-3’, ITS2: 5’-GCTGCGTTCTTCATCGATGC-3’) universal primers targeting the V3V4 region of the 16S rRNA gene (for bacteria) and the ITS2 region (for fungi). Absolute abundance measurements for **(A-B)** heads including mouthparts and **(C-D)** hindguts **(**replicated from Fig. 3C for visual comparison) from the same set of matched individuals. Data depicts the median (line in box), IQR (box), and minimum/maximum (whiskers) of n=33 distinct hives (n=7/7/7/6/6 for NTC/P/P+LX3/S/S+LX3 groups, respectively) across the six timepoints. Statistics shown for two-way ANOVA with BH-corrected multiple comparisons.

SUPPLEMENTARY FIGURE 10

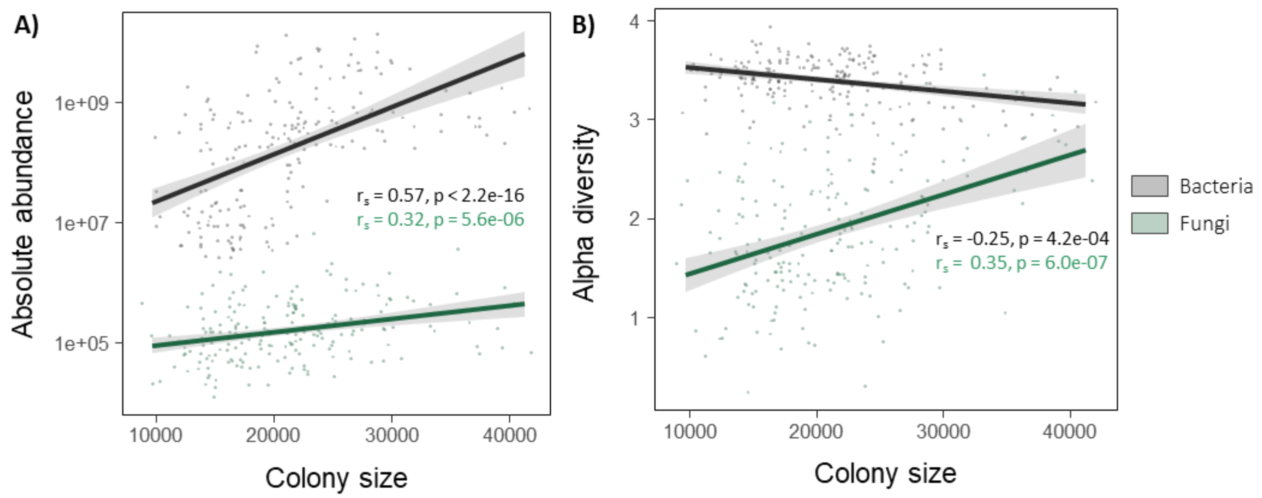

Supplementary Figure 10. Correlations between colony size and gut microbiota metrics. Scatterplots of colony size compared to **(A)** absolute abundance and **(B)** alpha diversity as measured by Shannon's H Index, for both bacterial (black) and fungal (green) comparisons. Data points represent n=3 pooled samples from a distinct hive at a distinct timepoint (N=594 total samples analyzed across 33 hives over 6 timepoints). Plots generated in R with regression lines computed via 'lm' method using the ggplot2 package and Spearman correlation statistics plotted with the stat_cor function of the ggpubr package.
